# Clinical study on single-port endoscopic resection via a gasless transaxillary approach in the treatment of breast fibroadenoma in adolescents

**DOI:** 10.1186/s12893-023-02186-1

**Published:** 2023-09-14

**Authors:** Jing-Yu Lu, Guo-Liang Zhang, Xiao-Jing Lin, Dar-Ren Chen, Zi-Fang Zheng, Yu Chen, Li-sheng Lin

**Affiliations:** 1 Department of Breast Surgery, The Affiliated Hospital of Putian University, 351100 Putian, Fujian China; 2 Department of Thyroid Surgery, The Affiliated Hospital of Putian University, 351100 Putian, Fujian China; 3 Operation Room, The Affiliated Hospital of Putian University, 351100 Putian, Fujian China; 4 Comprehensive Breast Cancer Centre, Changhua Christian Hospital, Changhua, Taiwan; 5 School of Clinical Medicine, Fujian Medical University, (No. 1 Xuefu North Road, University New District, 350122 Fuzhou, Fujian China

**Keywords:** Endoscopic surgery, Gasless method, Breast fibroadenoma in adolescents, Single-port, Transaxillary

## Abstract

**Background:**

Breast fibroadenoma is the most common benign breast tumour. This study aimed to investigate the advantages and disadvantages of endoscopic-assisted resection via a gas-less transaxillary single-port approach for breast fibroadenoma in adolescent patients, compared with a traditional approach.

**Methods:**

The clinical data of 83 patients with breast fibroadenoma treated in our hospital from October 2019 to October 2021 were collected for retrospective analysis. These patients were divided into an endoscopic-assisted surgery (ES) group (n = 39) and a traditional open surgery (OS) group (n = 44) according to the surgical approach. The operative time, intraoperative blood loss, incision length, postoperative complications, and patient satisfaction were compared between the two groups.

**Results:**

The surgical cost was (5.1 ± 0.6) thousand Yuan [(0.7 ± 0.1) thousand US dollars] in the ES group and (3.5 ± 2.7) thousand Yuan [(0.5 ± 0.4) thousand US dollars] in the OS group, showing a statistically significant difference (*p* < 0.001). There was no significant difference in surgical time, intraoperative blood loss, incision length, or the rate of postoperative complications between the two groups. Stratified analysis revealed that the ES group had a significantly shorter operative time [(57.00 ± 10.26) min vs. (78.27 ± 7.63)] (*p* < 0.001), a smaller incision length [(3.73 ± 0.34) cm vs. (4.42 ± 0.44) cm] (*p* < 0.001), and a lower complication incidence rate (11.1% vs. 63.6) (*p* = 0.011) than the OS group in the cases with a nodule number ≥ 3. The satisfaction score using the BREAST-Q scale indicated that psychosocial well-being and patient satisfaction with the breast in the ES group were significantly superior to those in the OS group [(91.18 ± 3.12) points vs. (87.00 ± 4.45) points and (91.03 ± 6.80) points vs. (84.45 ± 6.06) points, respectively] (*p* < 0.001).

**Conclusion:**

ES is a safe and effective method for the treatment of fibroadenoma. In patients with multiple fibroadenomas (≥ 3 tumours), ES has a shorter operative time and fewer postoperative complications. ES demonstrates a significant, prominent advantage in cosmetic appearance. However, it should be noted that ES is associated with higher costs than OS.

**Supplementary Information:**

The online version contains supplementary material available at 10.1186/s12893-023-02186-1.

## Background

Breast fibroadenoma, a mixed tumour derived from fibrous tissues and glandular epithelia of the breast, is the most common benign breast tumour, accounting for approximately 50% of all breast tumours [1–3]. This disease occurs in females of different ages, but adolescents are considered a high-risk group due to their active ovarian functions and high hormone levels during this period [4]. Breast fibroadenoma in adolescents mainly presents as a local breast mass with good mobility and a clear boundary. High levels of oestrogen and progesterone in adolescent females may result in breast fibroadenomas that rapidly increase in size. A few small nodules (< 1.0 cm in diameter) may not require surgical intervention, but single large masses (≥ 3 cm) or multiple large breast masses, which affect the appearance of the breast, need to be surgically resected [5]. A study reported by Hudson et al. [6] suggests surgical intervention for tumours larger than 2 cm in size. In 1998, Wang et al. [7] performed breast fibroadenoma resection using mastoscopy for the first time. Additionally, total endoscopy has also been applied to treat breast diseases in China, and it has achieved significant development to date [8].

Traditional surgery causes a relatively large wound and more complications in patients, resulting in a serious impact on the aesthetic appearance of the breast [9]. As the most representative minimally invasive technique, endoscopic-assisted surgery (ES) has been applied for resection of benign breast tumours in clinical practice with the extensive utilization of endoscopes and the development of minimally invasive techniques. In this study, ES was used to resect breast fibroadenomas and thus met the physical and psychological requirements of patients, which ultimately cured the disease and protected breast tissues and the nipple-areola complex to the greatest extent. Therefore, the present study explored the safety and efficacy of ES for the treatment of breast fibroadenoma in adolescents *via* comparisons between ES and open surgery (OS).

## Methods

### Patients

From October 2019 to October 2021, a total of 83 adolescent female patients (13–22 years old) diagnosed with breast fibroadenoma were enrolled in this study. The cohort was divided into an ES group (n = 39) and an OS group (n = 44) based on their surgical approaches, which were determined by the patients voluntarily. Their clinical data were collected for retrospective analysis [4].

The inclusion criteria were as follows: (1) adolescent female patients aged 13–22 years old who were (2) diagnosed with a breast fibroadenoma (≥ 3 cm in size) or multiple breast fibroadenomas (≥ 3 cm in maximum tumour diameter) confirmed by puncture pathology before the operation.

The exclusion criteria were (1) patients with coagulation abnormalities diagnosed by preoperative tests, (2) patients with breast cancer or sarcoma confirmed by pathological results, and (3) patients with a history of breast surgery.

Core needle biopsy was indicated in tumors larger than 5 cm or whenever the ultrasound BI-rads score is > 3 [10, 11].

Before the operation, the patients were examined by blood cell analysis, biochemical tests, coagulation tests [activated partial thromboplastin clotting time (APTT), prothrombin time (PT), fibrinogen (FIB), and thromboplastin time (TT)], pretransfusion immunity tests, and breast ultrasonography, and the tumours were confirmed to be breast fibroadenoma by puncture pathology preoperatively.

### Surgical procedure

Routine preoperative preparation was performed. Both groups of patients underwent general endotracheal anaesthesia, and the tumour location was routinely marked on the corresponding body surface before the operation.

In the OS group, the patient was in the supine position with 90° abduction of the upper limb on the affected side. A 2.0-5.5-cm arc incision was made adjacent to the areola (or an appropriate radial extension incision with an “Ω” shape along the arc incision according to the tumour size). The tissues were separated by an electric knife along the gland surface to the tumour surface and then radially separated, and after entering the gland, the tumour was identified and resected completely. Then, the incised end of the breast gland was sutured to prevent local collapse.

**Fig. 1:**
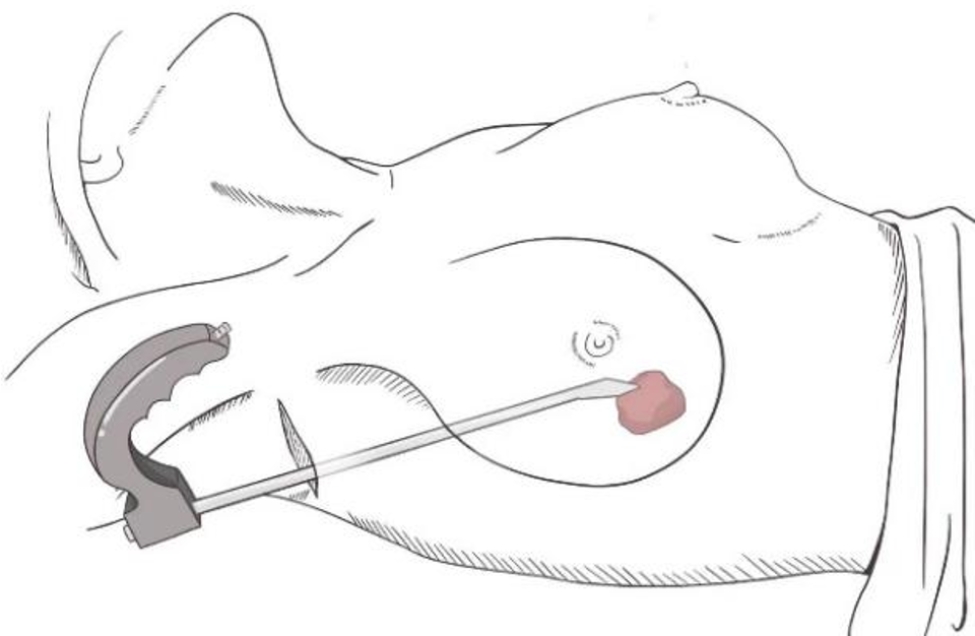
Schematic diagram of the patient’s position and endoscope placement

**Fig. 2:**
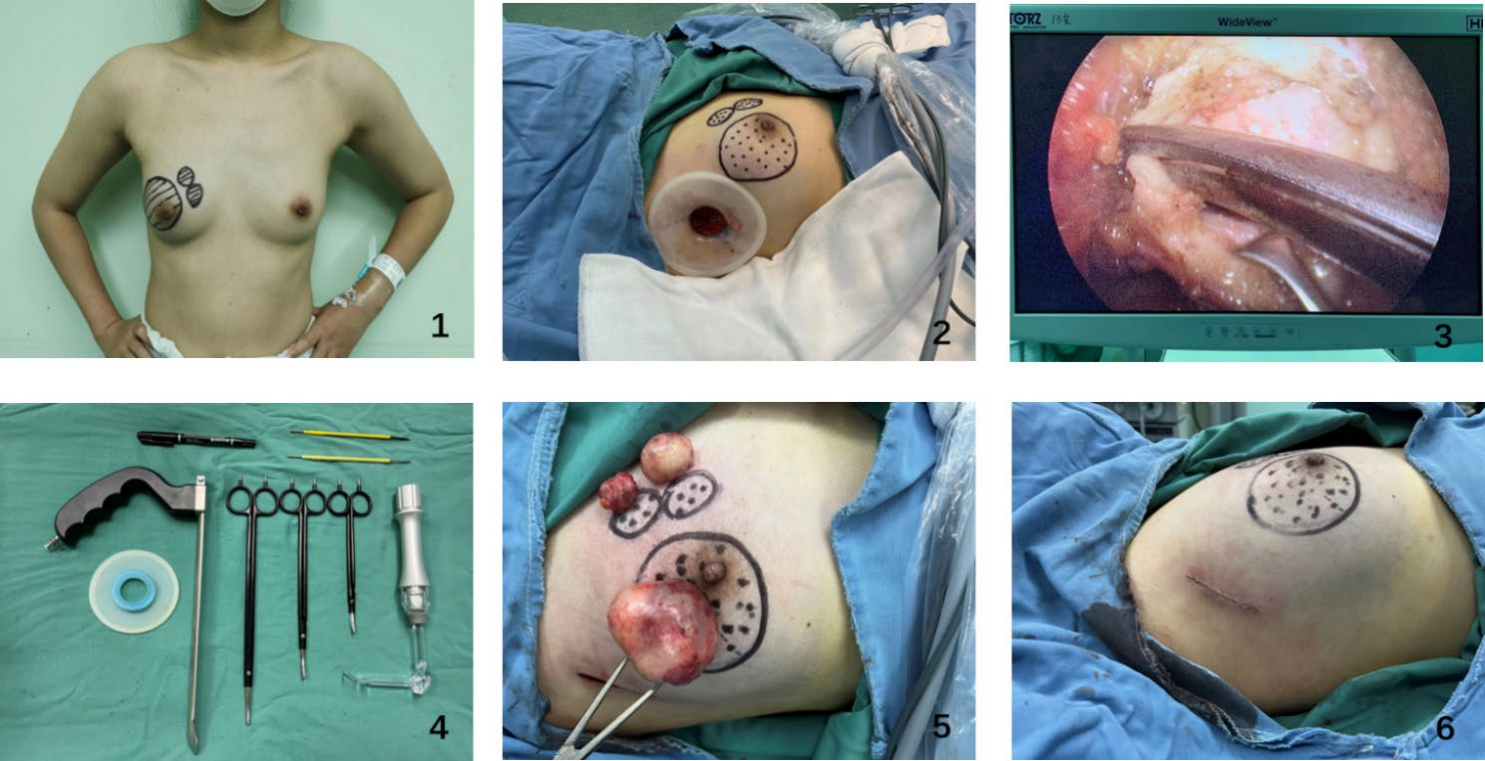
Procedures of endoscopic resection for breast fibroadenoma. (1) The tumour was located and marked on the body surface before the operation. (2) Creation of the front axillary incision and placement of the disposable incision protector. (3) Removal of the tumour and surrounding tissue by bipolar shearing. (4) Surgical instruments (endoscope, bipolar electric shear, light source pull hook, etc.) (5) Tumour in vitro. (6) Appearance after incision suture.

**Fig. 3:**
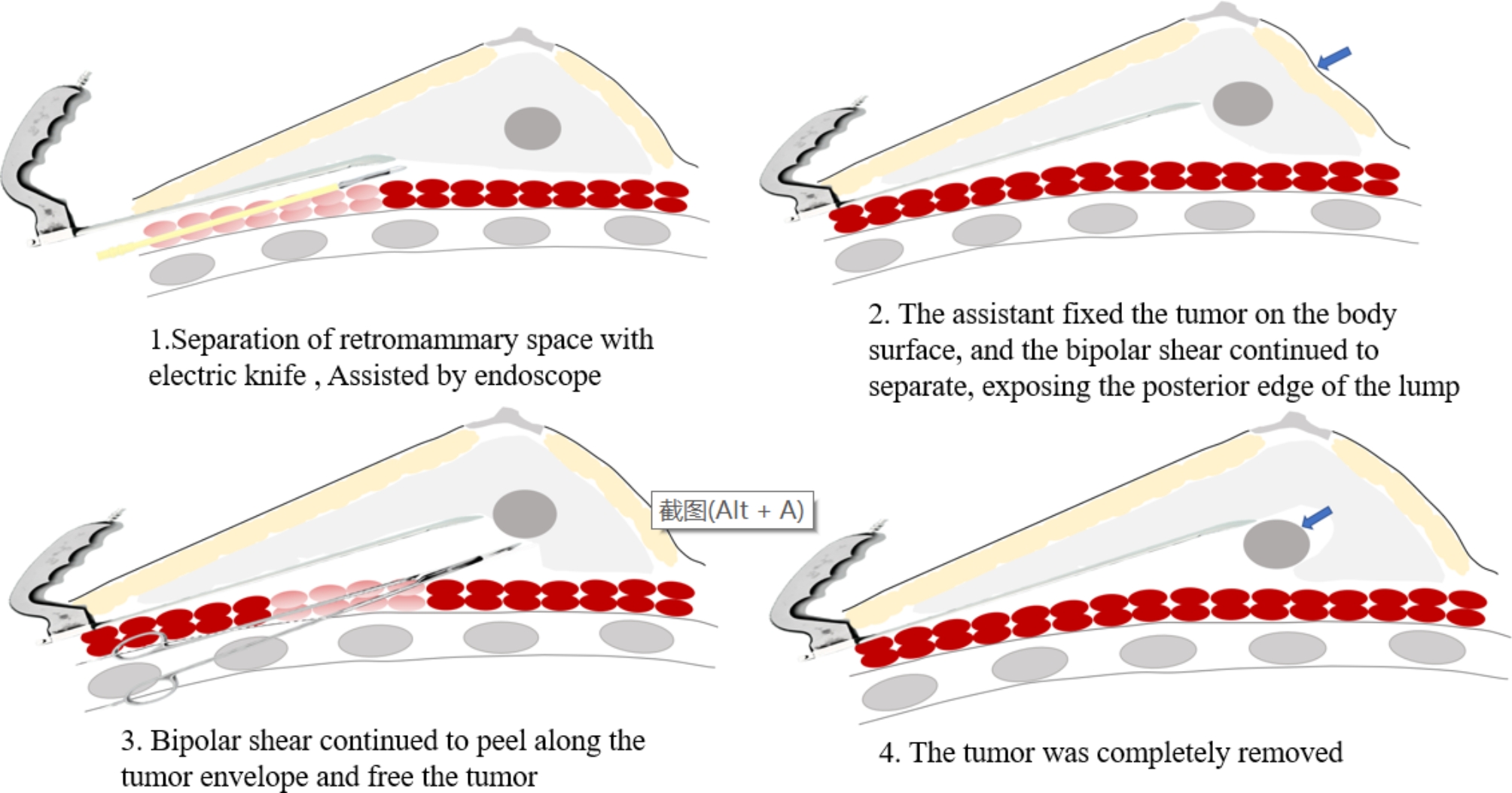
The surgical procedure of endoscopic resection is shown with a schematic diagram.

In the ES group, the patient was placed in the supine position with 90° abduction of the upper limb on the affected side. A 2.0-5.0-cm incision was made at the lateral anterior axillary line of the breast along the skin, and the skin and subcutaneous tissues were cut open and separated to the lateral edge of the pectoralis major muscle by an electric knife. Then, a disposable skin incision protector was placed at the incision, and an endoscope (Germany Karl Storz 49205FD) was inserted (Fig. 1). Under endoscopic guidance, the posterior space of the breast was further separated by an electric knife on the deep surface of the superficial fascia to reach the posterior side of the tumour. The assistant helped the surgeon fix the tumour in front of the endoscope lens. Next, bipolar electrosurgical scissors (Bipolar Scissor BP520/540/560) and dissecting forceps were used to separate normal glandular tissues around the tumour sharply and bluntly. The tumour capsule had to be intact without rupture to avoid tumour cell implantation in the surgical field. If necessary, a thick suture was passed through a small amount of normal tissue on the tumour surface for traction. Then, the tumour was completely resected. The surgical procedure of endoscopic resection is shown in Figs. 2 and 3. After complete haemostasis, the incision end of the breast tissues remaining after tumour resection was not sutured, and no drainage tube was needed.

The surgical procedures in both groups were performed by physicians on the same medical team. After the operation, compression bandaging with a cohesive elastic bandage was routinely applied in both groups.

### Observational indicators

The indicators including maximum tumour diameter, number of tumours, length of hospital stay, lesion site, distance from the centre of the nipple to the distal end of the largest tumour, incision length, operation time (i.e., the time from the start of the skin incision to the end of suturing), intraoperative blood loss (converted according to 1 g of blood ≈ 1 mL, blood loss volume = weight of small gauzes after the operation – weight of small gauzes before the operation; small gauzes were quantified by the weighing method to 0.1-g accuracy), surgical cost, and postoperative complications (e.g., subcutaneous ecchymosis, incision dehiscence, subcutaneous hydrops, and areola depression) were recorded. The Clavien‒Dindo classification system was used to grade the complications [12, 13]. All patients were followed up for at least 8 months. The BREAST Q questionnaire (Chinese translated version, Copyright of Memorial Sloan Kettering Cancer Center and The University of British Columbia) was administered to survey the patients’ physical well-being, psychosocial well-being, satisfaction with their breasts and medical experience.

Breast-Q 2.0 was translated, back translated and culturally debugged by Shanghai Fudan University Cancer Hospital after being authorized in writing by Memorial Sloan Caitlin Cancer Center (MSKCC) in 2017. After scoring according to the Qscore rule, an analysis of Cronbach’s reliability (Cronbach’s α coefficient), split-half reliability (Spearman-Brown coefficient), and structural validity was conducted for each module and dimension. It has been ultimately confirmed that the Chinese version of the Breast-Q questionnaire demonstrates good reliability and validity and is suitable for use in China [14, 15]. All BREAST-Q scores ranged from 0 to 100. Raw data were converted to a “Q-score” ranging from 0 to 100. A high score corresponded to a high health-related Quality of Life (QOL).

### Statistical methods and data analysis

SPSS 24.0 and R version 4.2.2 software were used for statistical analysis. The Kolmogorov‒Smirnov test was used to test the Gaussianity of the data, and *p* > 0.05 was considered to indicate conformation to a normal distribution. Data conforming to a normal distribution are expressed herein as (x̄±s) and were analysed by the independent-samples T test. Data that did not obey a normal distribution are expressed as the median (range); for these, the Mann‒Whitney U test was used for analysis. Enumeration data are expressed as numbers (%) and were analysed by the chi-square test or Fisher’s test. The significance level was α = 0.05; *p* < 0.05 was considered to indicate statistical significance.

## Results

### Basic data

The median age in the ES group was 18 years, ranging from 13 to 19 years, and that in the OS group was 18 years, ranging from 13 to 22 years. There were 93 tumours in the ES group and 88 tumours in the OS group. A total of 31 patients had tumour counts greater than or equal to 3, with 18 in the ES group and 13 in the OS group. The maximum tumour diameter was 3.64 ± 1.08 cm in the ES group and 3.69 ± 1.14 cm in the OS group. The distance from the centre of the nipple to the distal end of the tumour was 4.45 ± 2.40 cm in the ES group and 4.65 ± 2.43 cm in the OS group. The median tumour number was 2 (1–4) in both groups. There was a significant difference in tumour numbers between the two groups. No significant difference was observed in age, maximum tumour diameter, length of hospital stay, lesion site, or the distance from the centre of the nipple to the distal end of the largest tumour between the two groups (*p* > 0.05, Table 1).

**Table 1:** Comparison of the general data for patients between the ES group and OS group

	ES group (n = 39)	OS group (n = 44)	*u/t*/χ^2^	*P*
Age (y)	18(13–19)	18(13–22)	778	0.311
Maximum tumour diameter (cm)	3.64 ± 1.08	3.69 ± 1.14	-0.214	0.831
Number of tumours (n)	2(1–4)	2(1–4)	626.5	0.026
Postoperative hospital stay (h)	19.5(16–22)	19.8(16–22)	721	0.211
Distance from the centre of the nipple to the distal end of the tumour(cm)	4.45 ± 2.40	4.65 ± 2.43	-0.375	0.709
Lesion site, (n)			0.025	0.875
Left	17(43.6)	21(47.7)		
Right	22(56.4)	23(52.3)		
Lesion area, (n)			0.843	0.933
Upper outer quadrant	14(35.9)	13(29.5)		
Lower inner quadrant	3(8.7)	4(9)		
Upper inner quadrant	4(10.3)	7(15.9)		
Lower outer quadrant	5(12.8)	5(11.4)		
Multi quadrant	13(33.3)	15(34.1)		

ES: endoscopic surgery. OS: traditional open surgery. P < 0.05 was considered to indicate statistical significance

### Surgical data

All the tumours were completely removed, the operation went smoothly without any accidents, and none of the patients in the ES group were converted to open surgery. The intraoperative blood loss was 3.06 ± 2.41 ml in the ES group and 4.00 ± 3.42 ml in the OS group. The total incision length was 3.59 ± 0.40 cm in the ES group and 3.81 ± 0.80 cm in the OS group. The operative time was 45.54 ± 14.68 min in the ES group and 44.14 ± 22.31 min in the OS group. The surgical cost was 5.1 ± 0.6 thousand Yuan [(0.7 ± 0.1) thousand US dollars] in the ES group and 3.5 ± 2.7 thousand Yuan [(0.5 ± 0.4) thousand US dollars] in the OS group. The surgical cost in the ES group was significantly higher than that in the OS group (*p* < 0.001). There were no significant differences between the two groups in intraoperative blood loss (*p* = 0.149), incision length (*p* = 0.352) or operative time (*p* = 0.734) (Table 2).

**Table 2:** Comparison of surgical data between the two groups

	ES group (n = 39)	OS group (n = 44)	*t*/χ^2^	*P*
Intraoperative blood loss (mL)	3.06 ± 2.41	4.00 ± 3.42	-1.456	0.149
Total incision length (cm)	3.59 ± 0.40	3.81 ± 0.80	0.937	0.352
Operative time (min)	45.54 ± 14.68	44.14 ± 22.31	0.342	0.734
Surgical cost (thousand RBM)	5.1 ± 0.6	3.5 ± 2.7	15.82	< 0.001

ES: endoscopic surgery. OS: traditional open surgery. P < 0.05 was considered to indicate statistical significance

### Complications

In terms of overall complications, 1 (2.6%) case of subcutaneous ecchymosis, 1 (2.6%) case of poor healing of the incision (incision dehiscence), 1 (2.6%) case of subcutaneous hydrops, and 1 (2.6%) case of breast local depression were noted in the ES group, while 2 (4.5%) cases of subcutaneous ecchymosis, 2 (4.5%) cases of poor local flap healing of the areola incision, 4 (9.1%) cases of subcutaneous hydrops, and 5 (11.4%) cases of areola or breast local depression were observed in the OS group. All complications were classified as Clavien‒Dindo grade I. There were no statistically significant differences in the incidence rate (*p* = 0.057, Table 3) or the grades of complications (*p* = 0.054, Table 4) between the two groups. Incision dehiscence resolved after debridement and suture, subcutaneous hydrops was completely treated by puncture and suction supplemented with compression bandaging, and areola depression was ameliorated after local fat transplantation.

**Table 3:** Comparison of postoperative complications between the two groups

	ES group (n = 39)	OS group (n = 44)	χ^2^	*P*
Complications, n (%)				
Subcutaneous ecchymosis	1(2.6)	2(4.5)		
Incision dehiscence	1(2.6)	2(4.5)		
Subcutaneous hydrops	1(2.6)	4(9.1)		
Breast/areola depression	1(2.6)	5(11.4)		
Total	4(10.3)	13(29.5)	3.613	0.057

ES: endoscopic surgery. OS: traditional open surgery. P < 0.05 was considered to indicate statistical significance

**Table 4:** Clavien‒Dindo classification for postoperative complications

	ES group (n = 39)	OS group (n = 44)	χ^2^	P
Complication classification, n(%)			/	0.054
I	4(10.3)	13(29.5)		
II	0(0)	0(0)		
III	0(0)	0(0)		
IV	0(0)	0(0)		
V	0(0)	0(0)		

ES: endoscopic surgery. OS: traditional open surgery

### Stratified analysis

Patients were divided into two subgroups in both the ES and OS groups according to nodule number. Then, stratified comparisons were performed between the two groups. The results revealed that the ES group had a significantly shorter operative time [(57.00 ± 10.26) min vs. (78.27 ± 7.63) min] (*p* < 0.001) and a smaller incision length [(3.73 ± 0.34) cm vs. (4.42 ± 0.44) cm] than the OS group in cases with ≥ 3 nodules (*p* < 0.001) (Table 5).

**Table 5:** Stratified analysis of observational indicators in the two groups

	Number of tumours (< 3)		*p*	Number of tumours (≥ 3)		*p*
	Endoscopic group (n = 21)	Traditional open surgery group (n = 33)		Endoscopic group (n = 18)	Traditional open surgery group (n = 11)	
Intraoperative blood loss (mL)	3.00 ± 2.20	3.96 ± 3.55	0.270	3.13 ± 2.71	4.09 ± 3.13	0.388
Total Incision length (cm)	3.48 ± 0.42	3.61 ± 0.79	0.482	3.73 ± 0.34	4.42 ± 0.44	˂0.001
Operative time (min)	35.71 ± 10.04	32.76 ± 10.80	0.318	57.00 ± 10.26	78.27 ± 7.63	˂0.001
Surgical cost (ten thousand RMB)	0.54 ± 0.06	0.35 ± 0.03	0.000	0.48 ± 0.04	0.34 ± 0.03	˂0.001
Complications, n (%)	2(9.5)	6(18.2)	0.461	2(11.1)	7(63.6)	0.011
Subcutaneous ecchymosis	1(4.8)	1(3.0)		0(0)	1(9.1)	
Incision dehiscence	1(4.8)	1(3.0)		0(0)	1(9.1)	
Subcutaneous hydrops	0(0)	2(6.1)		1(5.6)	2(18.2)	
Areola depression	0(0)	2(6.1)		1(5.6)	3(27.3)	

ES: endoscopic surgery. OS: traditional open surgery. P < 0.05 was considered to indicate statistical significance

Subgroup analysis showed that in patients with fewer than three breast nodules, the differences in intraoperative blood loss, incision length, operative time, and postoperative complications were not significant between the ES group and the OS group, while 3 or more tumours were present, the operative time and total incision length in the ES group were significantly shorter than those in the OS group (*p* < 0.001), and the incidence rate of patients with postoperative complications in the ES group was significantly lower than that in the OS group (*p* = 0.011) (Table 5).

### Follow-up results

In addition, both groups of patients were followed up using the BREAST-Q scale from the U.S. for a period of 8–32 months until June 2022, with an average of 15.81 months. Postoperative psychosocial well-being was markedly elevated in both groups, but OS showed an impact on postoperative physical health, mainly discomfort in the areola area (*p* < 0.001). No significant difference was identified in preoperative BREAST-Q between the two groups (*p*_1_ > 0.05), and the postoperative follow-up indicated that psychological status was better and patient satisfaction with breast appearance was higher in the ES group than in the OS group (*p*_2_ < 0.001) (Table 6).

**Table 6:** Patient Satisfaction with the BREAST-Q

	Preoperative (1)	Preoperative (2)	*p* 1(Pre)	Postoperative (1)	Postoperative (2)	*p* 2 (Post)
Physical well-being	22.31 ± 2.53	22.16 ± 2.50	0.789	22.77 ± 2.64	24.00 ± 3.29	0.066
Sexual well-being	–	–	–	–	–	–
Psychosocial well-being	85.44 ± 4.09	84.84 ± 4.28	0.520	91.18 ± 3.12	87.00 ± 4.45	˂0.001
Satisfaction with breasts	91.41 ± 6.67	90.05 ± 5.31	0.303	91.05 ± 6.98	89.07 ± 5.20	0.143
Satisfaction with nipples (1–4)						
Satisfaction with height				1.36 ± 0.49	1.41 ± 0.50	0.645
Satisfaction with alignment				1.62 ± 0.78	1.41 ± 0.69	0.206
Satisfaction with shape				1.77 ± 0.78	1.80 ± 0.76	0.877
Satisfaction with look				1.41 ± 0.50	1.32 ± 0.47	0.390
Satisfaction with sensation				1.31 ± 0.47	1.36 ± 0.49	0.596
Satisfaction with outcome				91.03 ± 6.80	84.45 ± 6.06	˂0.001
Satisfaction with information				57.46 ± 1.40	57.55 ± 1.64	0.803
Satisfaction with surgeon				100(100–100)	100(100–100)	
Satisfaction with medical team				100(100–100)	100(100–100)	
Satisfaction with office staff				100(100–100)	100(100–100)	

(1) Represents the ES group, (2) represents the OS group

The patients in the two groups were between 13 and 22 years old. After investigation, they had almost no sexual history, so they did not answer the relevant questions in the questionnaire. In the information project, because the study did not perform total mastectomy or reconstruction, related problems such as breast symmetry were also excluded

## Discussion

In this study, we surveyed the clinical characteristics and pros and cons of ES for the treatment of fibroadenoma of the breast by comparing them with OS and found that ES has a similar safety to that of OS but has a better cosmetic appearance than OS for the resection of fibroadenoma. ES is superior to OS in treating patients with 3 or more tumours, with a shorter operative time.

Operating time is one of the common indices used to record a surgery, which usually represents the complexity of the surgery. In this study, the operative time in the ES group (average 45.54 min) was slightly longer than that in the OS group. The mean time required for endoscopy in this study was 45.54 min, which was shorter than the average time required for three-port gas-inflation endoscopy (61.4 min) reported by Mlees et al. [16]. Although the advantages of ES in terms of operative time were not evident in this study, in the subgroup analysis, the operative time in the ES group was significantly and highly favourable when three or more tumours were present, suggesting that ES was easier to remove from multiple tumours than OS. The probable reason is that the endoscopic retractor and the cap-shaped structure at the front end of the endoscope can expand the surgical space easily, and vessels in the surgical field can be clearly visualized by the amplification of the endoscope, which helps prevent intraoperative bleeding. This enables quick identification and resection of lesions under the supervision of the high-definition endoscope.

The incidence of postoperative complications was 10.3% in the ES group and 29.5% in the OS group. All complications were mild and well resolved. There was no significant difference between ES and OS in terms of complication incidence. Lai et al. [17] reported an incidence of complications of 6.5% in a recent study, lower than our study, which was considered to be related to the sample size. Stratified analysis revealed that the ES group had a significantly lower complication incidence rate than the OS group in cases with nodule numbers ≥ 3. Our study suggests that gasless ES is much safer than OS in patients with multiple breast nodules.

In addition, ES has the following advantages over OS: (1) Endoscope-assisted resection of tumours through the posterior space of the breast can minimize electric heat damage to the subcutaneous fat layer of the gland surface, thus reducing the possibility of fat necrosis on the breast surface. (2) The application of an endoscope can avoid the damage to mammary ducts in the central area caused by traditional surgery through a para-areolar approach, leading to a reduced potential risk of mastitis in patients during lactation in the future. (3) Excessive traction on the flap inevitably occurs during the establishment of the surgical “tunnel” with a para-areolar approach, resulting in disturbance of the blood supply around the flap and causing ischaemic necrosis or deformation of the flap. This problem can be avoided during ES since the tumour is removed through the posterior breast space. (4) ES causes less damage to normal breast tissues in the superficial layer of a tumour, thereby reducing the likelihood of local collapse after tumour resection, which is rarely observed in the ES group. (5) The learning curve for ES is short. According to the authors’ clinical experience, the endoscopic method can be proficiently mastered by a surgeon who has completed the operation on five patients, with no difference in postoperative recovery time compared to OS. The endoscope has an integrated hook, a suction device, and a lens, and only the lens body and electrical output instruments enter the surgical cavity, which diminishes interference from various instruments in the surgical cavity with surgical procedures. Additionally, compared to traditional gas-inflation total endoscopy, the gasless method for cavity establishment can prevent the occurrence of subcutaneous emphysema and hypercapnia resulting from carbon dioxide retention caused by the gas-inflation method [16, 18]. Moreover, the endoscope can be operated by a single person, reducing or even eliminating the need for an endoscope holder. The surgical costs in the ES group increased by 45%, and a certain learning curve was required (at least 5 cases).

Mlees et al. [16] conducted endoscopic surgery to treat benign breast tumours, including resection of multiple tumours, and more than 80% of patients were satisfied with the aesthetic appearance of the breast after the operation. Studies have demonstrated that an areolar incision is more prone to scar hyperplasia than an axillary incision [19–21]. In this study, the incision in the ES group was made in the anterior axillary line close to the axillary fossa, which was hidden in the skin texture and relatively undetectable. Since the study population consisted of adolescent females, most of whom were unmarried, they were more concerned about the cosmetic appearance of the breast than other age groups. When choosing surgical approaches, they were more likely to choose ES due to its more covert incision. In this sense, adolescent female patients are more suitable for ES [22].

The BREAST Q score is a scale used to evaluate the health-related quality of life and satisfaction of patients undergoing breast surgery [23]. In this study, we applied this method to evaluate patients’ satisfaction with their breast and medical experiences. Patient satisfaction with the concealed axillary incision was markedly higher than that with a traditional para-areolar incision according to the satisfaction survey distributed during the postoperative follow-up. In terms of psychosocial well-being and patient satisfaction with the breast, the BREAST-Q scores were 91.18 ± 3.12 points and 91.03 ± 6.80 points, respectively, in the ES group, which were higher than the scores of 87.00 ± 4.45 points and 84.45 ± 6.06 points in the OS group, respectively, with statistically significant differences (*p* < 0.05). Based on these data, endoscope-assisted tumour resection is suitable for adolescent patients with breast fibroadenoma and is particularly advantageous for resection of multiple fibroadenomas.

Nonetheless, the endoscopic technique has some limitations. Our study did not include patients of other races, so further validation is needed to determine whether this technique can be applied to other racial groups. Although patient satisfaction improved, this does not negate the fact that the surgical cost was higher than that of the OS group. In addition, our small sample size may lead to research bias.

## Conclusions

Endoscope-assisted tumour resection is suitable for adolescent patients with breast fibroadenoma. However, larger studies and longer follow-up periods are needed to obtain more reliable postoperative results.

### Electronic supplementary material

Below is the link to the electronic supplementary material.

Supplementary Material 1

Supplementary Material 2

Supplementary Material 3

Supplementary Material 4

Supplementary Material 5

Supplementary Material 6

Supplementary Material 7

Supplementary Material 8

Supplementary Material 9

Supplementary Material 10

Supplementary Material 11

Supplementary Material 12

## Data Availability

The datasets created and/or analysed during the current study are available from the corresponding author upon reasonable request.

## Abbreviations

ES: endoscopic assisted surgery
OS: open surgery
MSKCC: Memorial Sloan Caitlin Cancer Center
QOL: Quality of Life
