# Supplementary figures and images for: Clinical study on single-port endoscopic resection via a gasless transaxillary approach in the treatment of breast fibroadenoma in adolescents
Source: BMC Surg. 2023 Sep 14;23:279. doi: 10.1186/s12893-023-02186-1 (PMC10503113; doi:10.1186/s12893-023-02186-1)

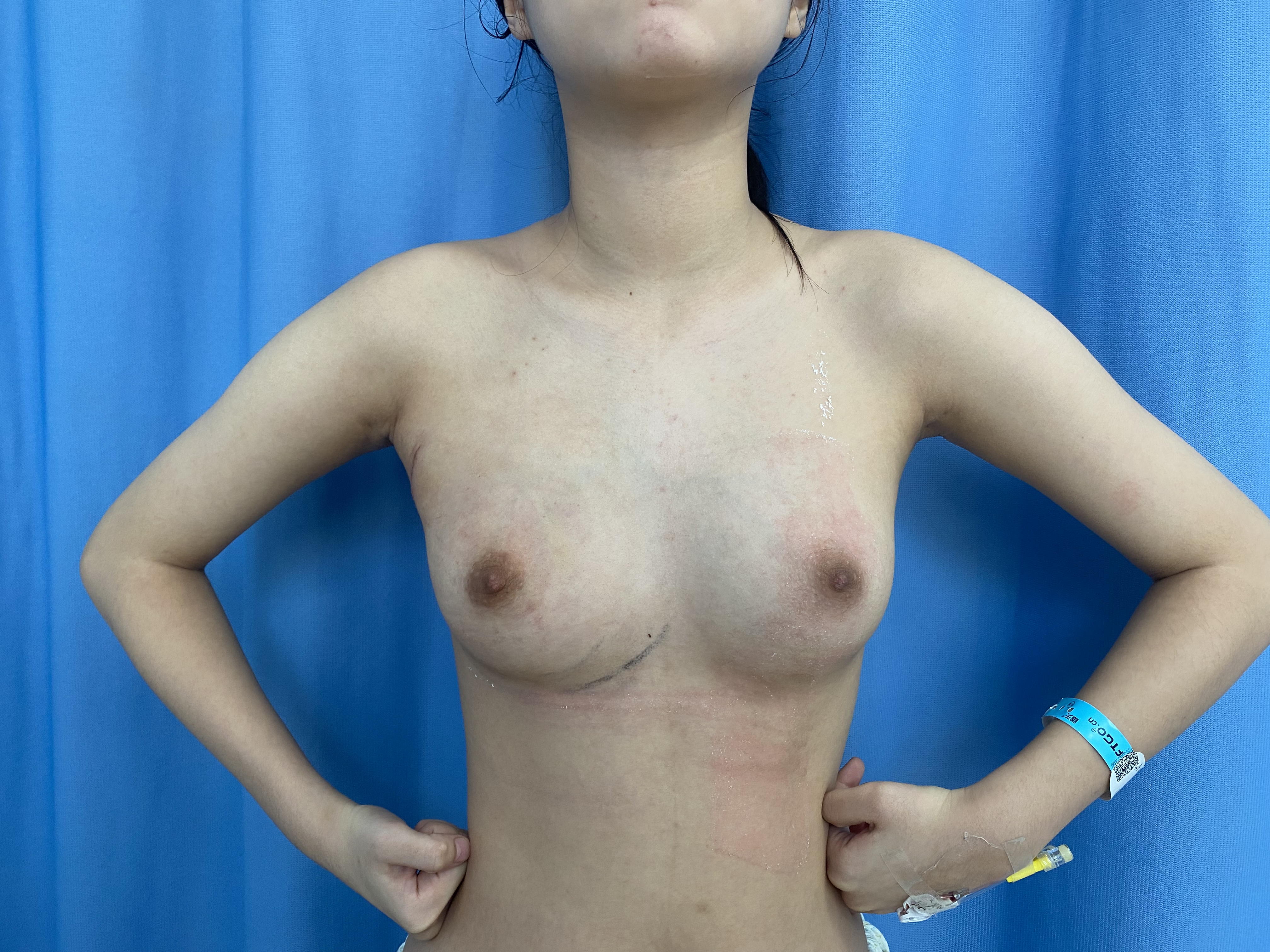

Supplement: Supplementary file 1 — Supplementary Material 1 [file 12893_2023_2186_MOESM1_ESM.jpg]

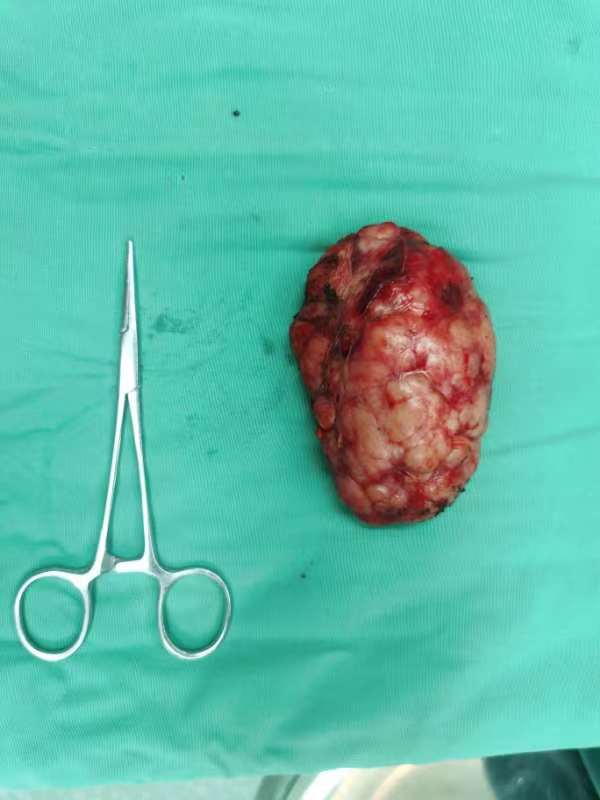

Supplement: Supplementary file 2 — Supplementary Material 2 [file 12893_2023_2186_MOESM2_ESM.jpg]

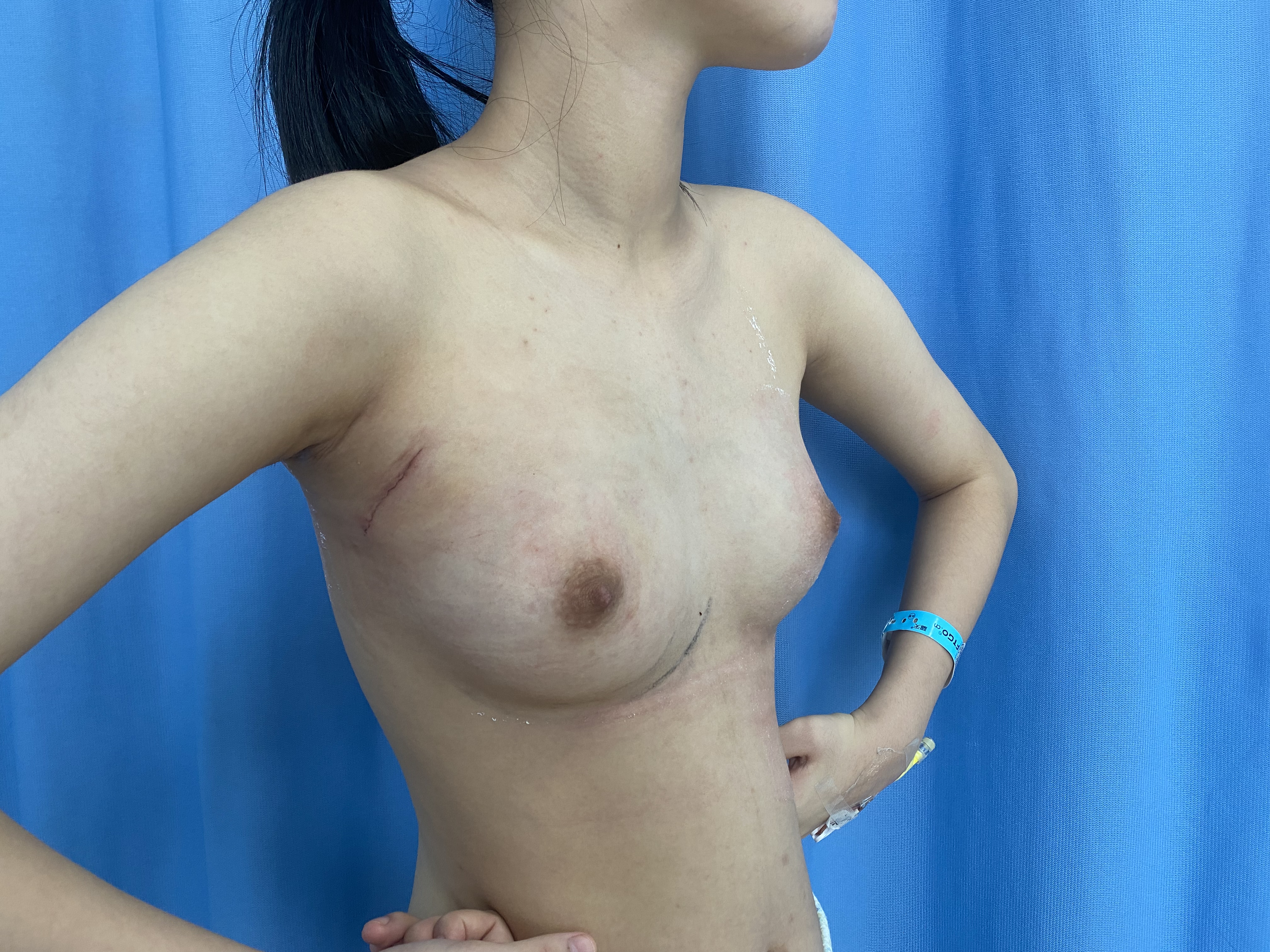

Supplement: Supplementary file 3 — Supplementary Material 3 [file 12893_2023_2186_MOESM3_ESM.jpg]

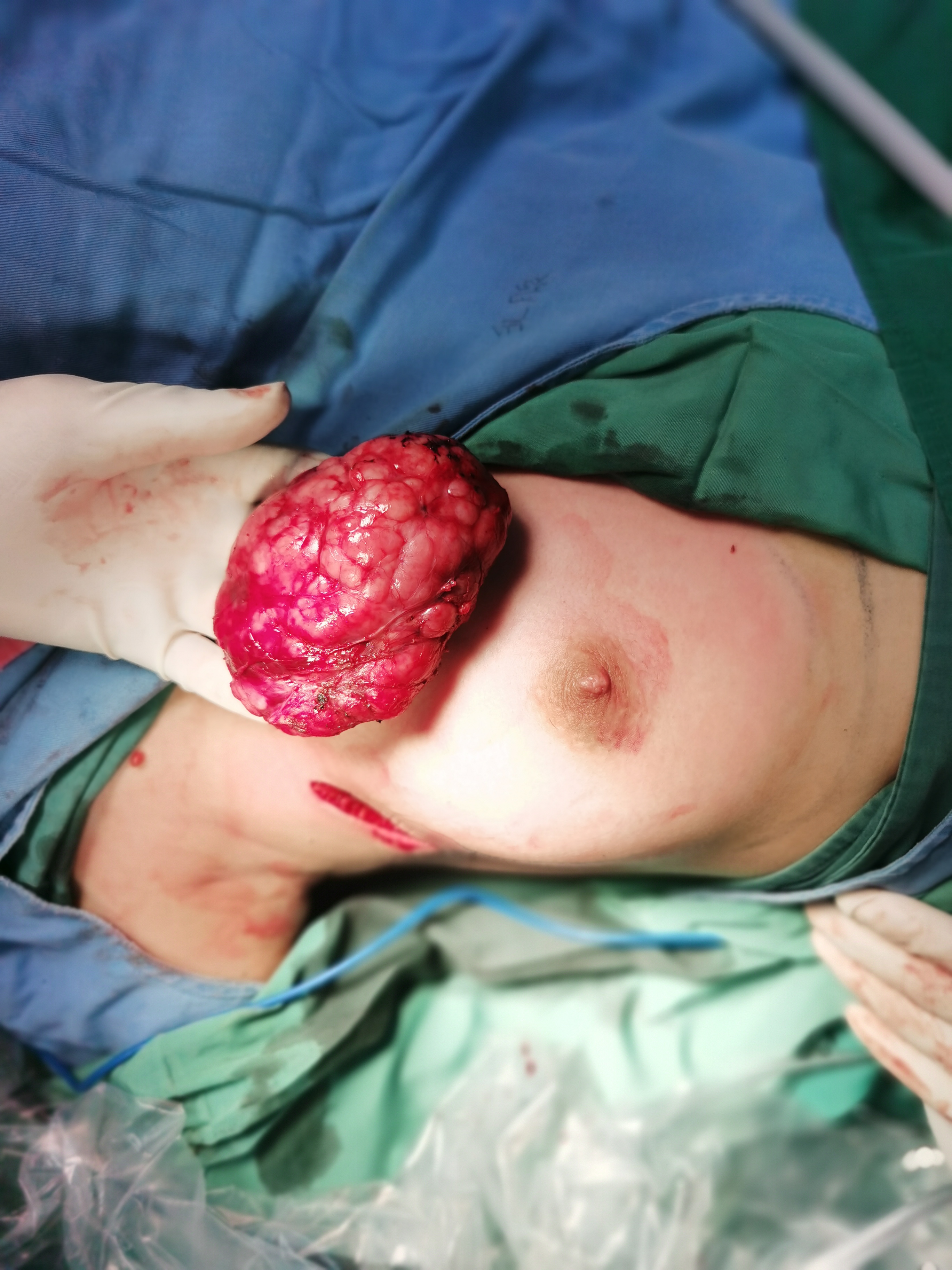

Supplement: Supplementary file 5 — Supplementary Material 5 [file 12893_2023_2186_MOESM5_ESM.jpg]

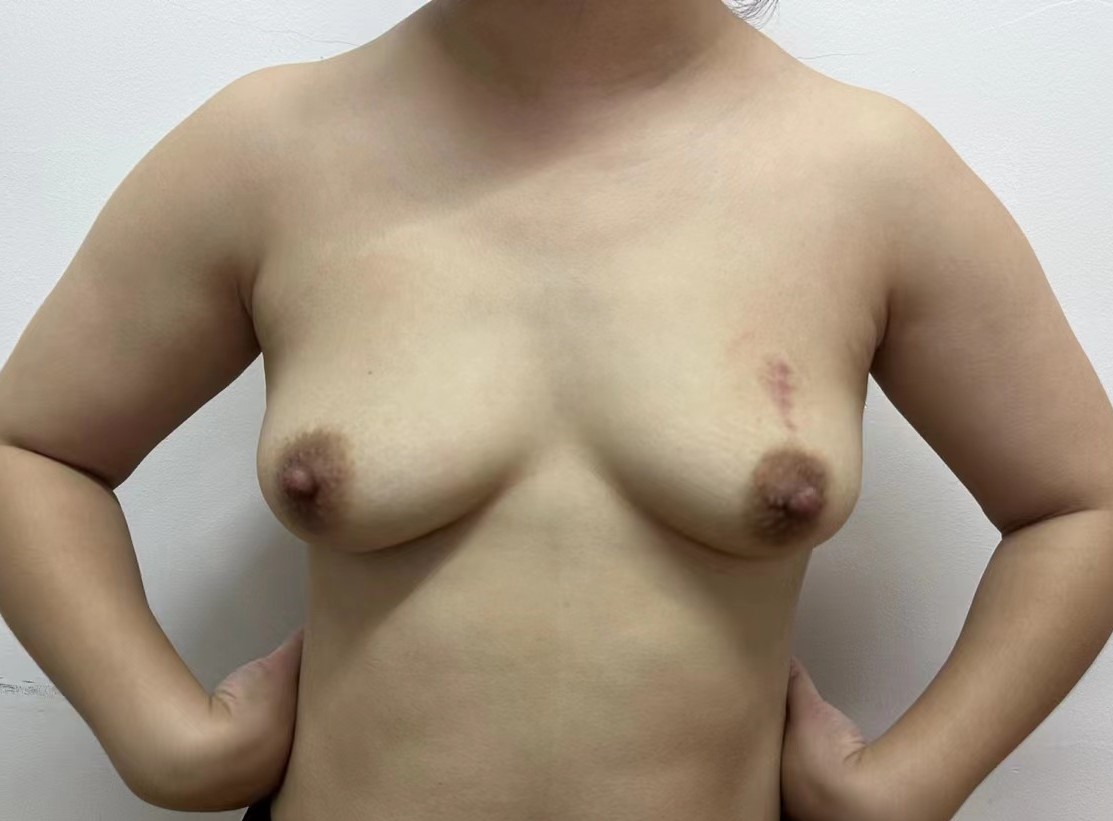

Supplement: Supplementary file 7 — Supplementary Material 7 [file 12893_2023_2186_MOESM7_ESM.jpg]

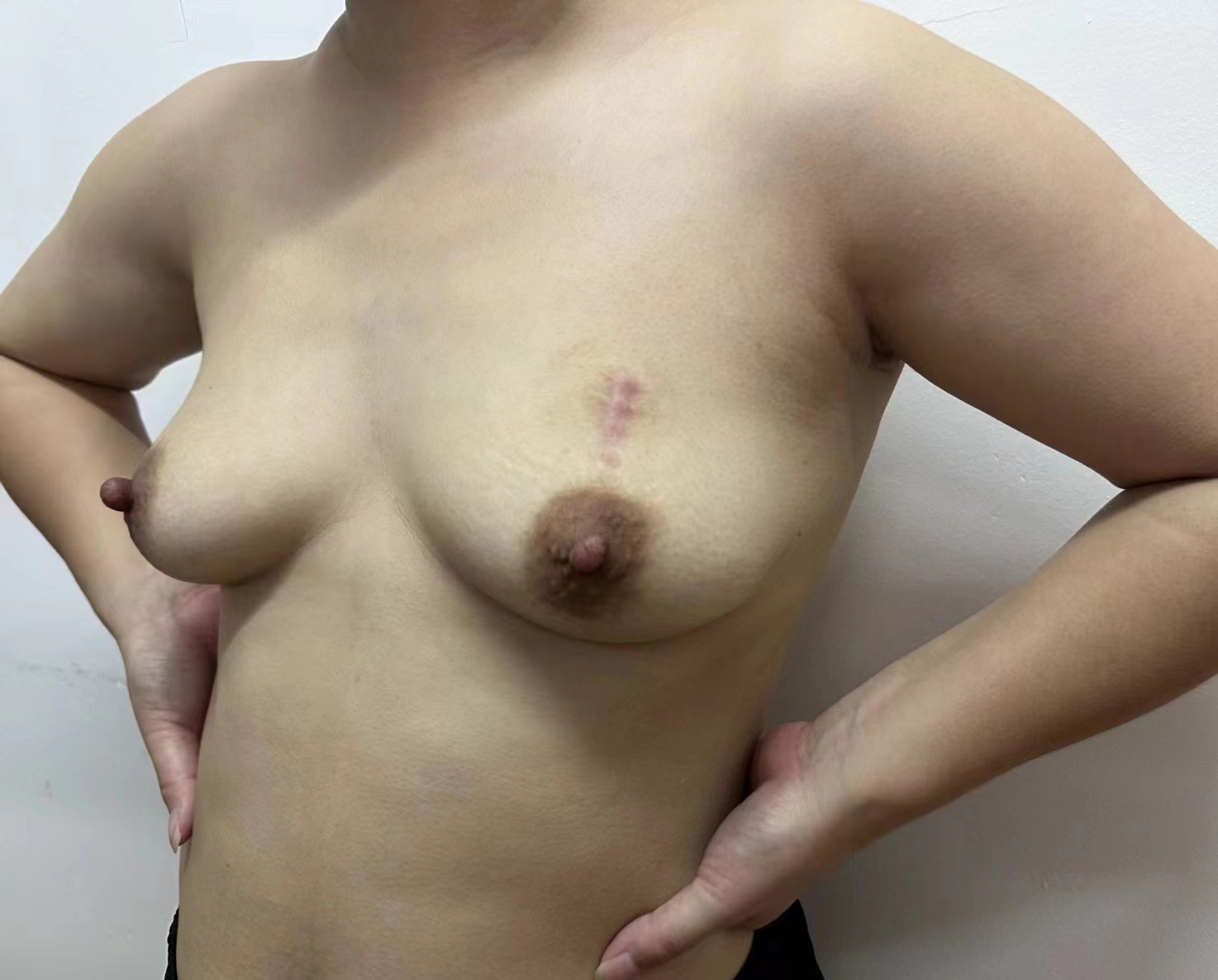

Supplement: Supplementary file 8 — Supplementary Material 8 [file 12893_2023_2186_MOESM8_ESM.jpg]

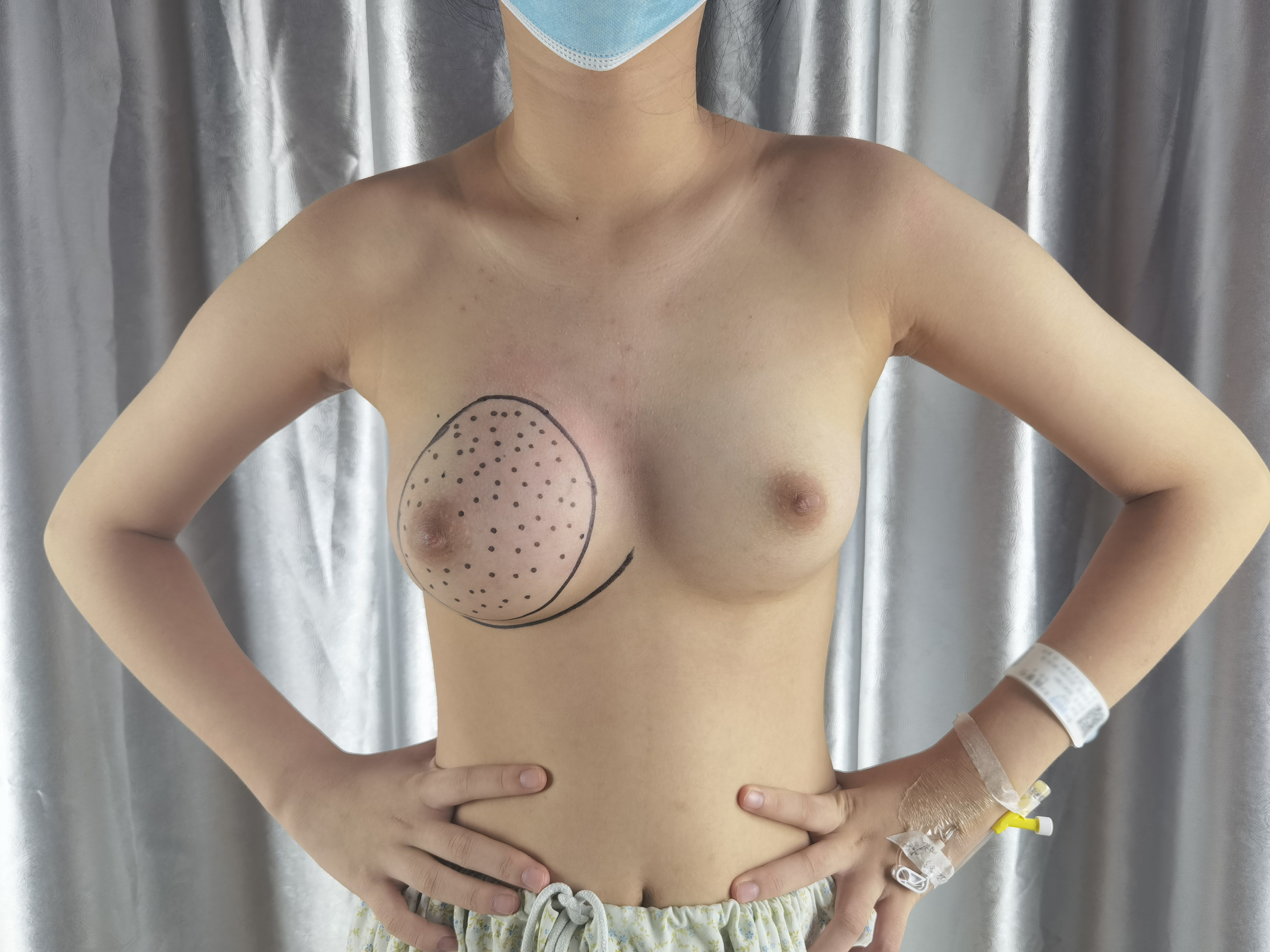

Supplement: Supplementary file 11 — Supplementary Material 11 [file 12893_2023_2186_MOESM11_ESM.jpg]
