# Supplementary material for: Clinical study on single-port endoscopic resection via a gasless transaxillary approach in the treatment of breast fibroadenoma in adolescents
Source: BMC Surg. 2023 Sep 14;23:279. doi: 10.1186/s12893-023-02186-1 (PMC10503113; doi:10.1186/s12893-023-02186-1)
Supplement: Supplementary file 4 — Supplementary Material 4 [file 12893_2023_2186_MOESM4_ESM.pdf]

Memorial Sloan Kettering Cancer Center

BREAST-Q Version 2.0©

Reduction/Mastopexy Module

Pre- and Postoperative Scales

Mandarin (CN) Version

THE UNIVERSITY OF BRITISH COLUMBIA

Translated by a local academic.

The BREAST-Q, authored by Drs. Andrea Pusic, Anne Klassen and Stefan Cano, is the copyright of Memorial Sloan Kettering Cancer Center and The University of British Columbia (Copyright ©2017, Memorial Sloan Kettering Cancer Center and the University of British Columbia). The BREAST-Q has been provided under license from Memorial Sloan Kettering Cancer Center and must not be copied, distributed or used in any way without the prior consent of Memorial Sloan Kettering Cancer Center.

BREAST-Q™ - REDUCTION MODULE (PRE- AND POSTOPERATIVE) VERSION 2.0: PSYCHOSOCIAL WELL-BEING

根据您乳房的情况，在过去的 1 周里，您是否有过以下经历：

	从没有	很少有	偶尔有	经常有	一直有
a. 在社交场合觉得有信心。	1	2	3	4	5
b. 觉得自己和其他女人一样的正常。	1	2	3	4	5
c. 自我感觉良好。	1	2	3	4	5
d. 觉得自信。	1	2	3	4	5
e. 对穿着打扮感到自信。	1	2	3	4	5
f. 认可自己的身材。	1	2	3	4	5
g. 外貌与内在相称。	1	2	3	4	5
h. 对自己的身材有信心。	1	2	3	4	5
i. 有吸引力。	1	2	3	4	5

BREAST-Q® VERSION 2.0 © Memorial Sloan Kettering Cancer Center and The University of British Columbia, 2017, All rights reserved

---

Note to Investigators: This scale can be used independently of the other scales.

The BREAST-Q, authored by Drs. Andrea Pusic, Anne Klassen and Stefan Cano, is the copyright of Memorial Sloan Kettering Cancer Center and The University of British Columbia (Copyright ©2017, Memorial Sloan Kettering Cancer Center and the University of British Columbia). The BREAST-Q has been provided under license from Memorial Sloan Kettering Cancer Center and must not be copied, distributed or used in any way without the prior consent of Memorial Sloan Kettering Cancer Center.

BREAST-Q™ - REDUCTION MODULE (PRE- AND POSTOPERATIVE) VERSION 2.0:
PSYCHOSOCIAL WELL-BEING CONVERSION TABLE

Instructions: If missing data is less than 50% of the scale's items, insert the mean of the completed items. Use the Conversion Table below to convert the raw scale summed score into a score from 0 (worst) to 100 (best). Higher scores reflect a better outcome.

SUM SCORE	EQUIVALENT RASCH TRANSFORMED SCORE (0-100)
9	0
10	14
11	18
12	21
13	24
14	26
15	28
16	30
17	32
18	33
19	35
20	36
21	38
22	39
23	41
24	42
25	44
26	45
27	47
28	49
29	50
30	52
31	54
32	56
33	59
34	61
35	64
36	66
37	69
38	72
39	75
40	78
41	81
42	84
43	88
44	93
45	100

BREAST-Q™ - REDUCTION MODULE (PRE- AND POSTOPERATIVE) VERSION 2.0: SEXUAL WELL-BEING

在性的方面，您是否有以下感觉：

	从没有	很少有	偶尔有	经常有	一直有
a. 在性行为中觉得舒适。	1	2	3	4	5
b. 对性生活有自信 。	1	2	3	4	5
c. 对性生活很满意。	1	2	3	4	5
d. 打扮后挺性感的。	1	2	3	4	5
e. 不穿衣服时挺性感的。	1	2	3	4	5

BREAST-Q® VERSION 2.0 © Memorial Sloan Kettering Cancer Center and The University of British Columbia, 2017, All rights reserved

Note to Investigators: This scale can be used independently of the other scales. The following statement can be added to the stem to provide an opportunity for the patient to decline completing this scale. 'The following questions ask about your sexual well-being. If you are uncomfortable answering these questions or do not feel that they apply to you, please check the box and skip the questions that follow.'

The BREAST-Q, authored by Drs. Andrea Pusic, Anne Klassen and Stefan Cano, is the copyright of Memorial Sloan Kettering Cancer Center and The University of British Columbia (Copyright ©2017, Memorial Sloan Kettering Cancer Center and the University of British Columbia). The BREAST-Q has been provided under license from Memorial Sloan Kettering Cancer Center and must not be copied, distributed or used in any way without the prior consent of Memorial Sloan Kettering Cancer Center.

BREAST-Q™ - REDUCTION MODULE (PRE- AND POSTOPERATIVE) VERSION 2.0: SEXUAL WELL-BEING CONVERSION TABLE

Instructions: If missing data is less than 50% of the scale's items, insert the mean of the completed items. Use the Conversion Table below to convert the raw scale summed score into a score from 0 (worst) to 100 (best). Higher scores reflect a better outcome.

SUM SCORE	EQUIVALENT RASCH TRANSFORMED SCORE (0-100)
5	0
6	18
7	23
8	28
9	31
10	34
11	37
12	39
13	42
14	44
15	47
16	50
17	53
18	56
19	60
20	65
21	71
22	76
23	82
24	90
25	100

BREAST-Q™ - REDUCTION MODULE (PRE- AND POSTOPERATIVE) VERSION 2.0: PHYSICAL WELL-BEING

在过去 1 周里，您是否有过以下经历：

	从没有	偶尔有	一直有
a. 头疼。	1	2	3
b. 乳房疼痛。	1	2	3
c. 精力不济。	1	2	3
d. 难以进行剧烈的体育活动（如：跑步或做操）。	1	2	3
e. 感觉身体不能保持平衡。	1	2	3
f. 肩膀疼。	1	2	3
g. 胸部感觉不适以至于影响睡眠。	1	2	3
h. 颈部疼痛。	1	2	3
i. 肩带造成的肩部勒痕及疼痛。	1	2	3
j. 感觉到身体上的不舒服。	1	2	3
k. 乳房上有皮疹。	1	2	3
l. 背部疼。	1	2	3
m. 上臂疼。	1	2	3
n. 因为乳房过大导致手疼痛、麻木或刺痛。	1	2	3

BREAST-Q® VERSION 2.0 © Memorial Sloan Kettering Cancer Center and The University of British Columbia, 2017, All rights reserved

Note to Investigators: This scale can be used independently of the other scales.

The BREAST-Q, authored by Drs. Andrea Pusic, Anne Klassen and Stefan Cano, is the copyright of Memorial Sloan Kettering Cancer Center and The University of British Columbia (Copyright ©2017, Memorial Sloan Kettering Cancer Center and the University of British Columbia). The BREAST-Q has been provided under license from Memorial Sloan Kettering Cancer Center and must not be copied, distributed or used in any way without the prior consent of Memorial Sloan Kettering Cancer Center.

BREAST-Q™ - REDUCTION MODULE (PRE- AND POSTOPERATIVE) VERSION 2.0: PHYSICAL WELL-BEING CONVERSION TABLE

Instructions: Items ‘a’ and ‘b’ are stand-alone items that are not included in the scale score. Recode items c, d, e, f, g, h, i, j, k, l, m, and n as follows: “None of the time” = 3; “Some of the time” = 2; “All of the time” = 1. If missing data is less than 50% of the scale’s items, insert the mean of the completed items. Use the Conversion Table below to convert the raw scale summed score into a score from 0 (worst) to 100 (best). Higher scores reflect a <u>better outcome</u>.

SUM SCORE	EQUIVALENT RASCH TRANSFORMED SCORE (0-100)
12	0
13	14
14	20
15	25
16	28
17	31
18	34
19	37
20	40
21	42
22	44
23	47
24	49
25	51
26	54
27	56
28	59
29	62
30	65
31	68
32	72
33	77
34	82
35	90
36	100

BREAST-Q^{TM} - REDUCTION MODULE (PREOPERATIVE) VERSION 2.0: SATISFACTION WITH BREASTS

根据您乳房的情况，在过去的 1 **周里**，**您**对以下情况是否满意：

	非常不满意	比较不满意	比较满意	非常满意
a. 穿衣服时胸部外形看起来如何？	1	2	3	4
b. 乳房大小和身材相称吗？	1	2	3	4
c. 乳房的大小如何？	1	2	3	4
d. 穿胸罩时乳房的形状如何？	1	2	3	4
e. 两侧的乳房大小一样吗？	1	2	3	4
f. 穿胸罩时舒服吗？	1	2	3	4
g. 没穿胸罩时乳房的形状如何？	1	2	3	4
h. 穿衣服时整体看起来如何？	1	2	3	4
i. 乳房的位置如何？	1	2	3	4
j. 乳房看起来正常吗？	1	2	3	4
k. 没穿衣服时整体看起来如何？	1	2	3	4

BREAST-Q® VERSION 2.0 © Memorial Sloan Kettering Cancer Center and The University of British Columbia, 2017, All rights reserved

Note to Investigators: This scale can be used independently of the other scales.

The BREAST-Q, authored by Drs. Andrea Pusic, Anne Klassen and Stefan Cano, is the copyright of Memorial Sloan Kettering Cancer Center and The University of British Columbia (Copyright ©2017, Memorial Sloan Kettering Cancer Center and the University of British Columbia). The BREAST-Q has been provided under license from Memorial Sloan Kettering Cancer Center and must not be copied, distributed or used in any way without the prior consent of Memorial Sloan Kettering Cancer Center.

BREAST-Q™ - REDUCTION MODULE (PREOPERATIVE) VERSION 2.0: SATISFACTION WITH BREASTS CONVERSION TABLE

Instructions: If missing data is less than 50% of the scale's items, insert the mean of the completed items. Use the Conversion Table below to convert the raw scale summed score into a score from 0 (worst) to 100 (best). Higher scores reflect a better outcome.

SUM SCORE	EQUIVALENT RASCH TRANSFORMED SCORE (0-100)
11	0
12	11
13	17
14	21
15	24
16	26
17	29
18	31
19	33
20	35
21	36
22	38
23	40
24	41
25	43
26	45
27	46
28	48
29	50
30	52
31	53
32	55
33	57
34	59
35	61
36	63
37	66
38	68
39	71
40	74
41	78
42	82
43	89
44	100

BREAST-Q™ - REDUCTION MODULE (POSTOPERATIVE) VERSION 2.0:
SATISFACTION WITH BREASTS

根据您乳房的情况，在过去的 1 周里，您对以下情况是否满意：

	非常不满意	比较不满意	比较满意	非常满意
a. 穿衣服时胸部外形看起来如何？	1	2	3	4
b. 乳房大小和身材相称吗？	1	2	3	4
c. 乳房的大小如何？	1	2	3	4
d. 穿胸罩时乳房的形状如何？	1	2	3	4
e. 两侧的乳房大小一样吗？	1	2	3	4
f. 穿胸罩时舒服吗？	1	2	3	4
g. 没穿胸罩时乳房的形状如何？	1	2	3	4
h. 穿衣服时整体看起来如何？	1	2	3	4
i. 乳房的位置如何？	1	2	3	4
j. 乳房看起来正常吗？	1	2	3	4
k. 瘢痕的位置如何？	1	2	3	4
l. 瘢痕看起来如何？	1	2	3	4
m. 没穿衣服时整体看起来如何？	1	2	3	4

BREAST-Q® VERSION 2.0 © Memorial Sloan Kettering Cancer Center and The University of British Columbia, 2017, All rights reserved

Note to Investigators: This scale can be used independently of the other scales.

The BREAST-Q, authored by Drs. Andrea Pusic, Anne Klassen and Stefan Cano, is the copyright of Memorial Sloan Kettering Cancer Center and The University of British Columbia (Copyright ©2017, Memorial Sloan Kettering Cancer Center and the University of British Columbia). The BREAST-Q has been provided under license from Memorial Sloan Kettering Cancer Center and must not be copied, distributed or used in any way without the prior consent of Memorial Sloan Kettering Cancer Center.

BREAST-Q™ - REDUCTION MODULE (POSTOPERATIVE) VERSION 2.0: SATISFACTION WITH BREASTS CONVERSION TABLE

Instructions: If missing data is less than 50% of the scale's items, insert the mean of the completed items. Use the Conversion Table below to convert the raw scale summed score into a score from 0 (worst) to 100 (best). Higher scores reflect a better outcome.

SUM SCORE	EQUIVALENT RASCH TRANSFORMED SCORE (0-100)
13	0
14	11
15	16
16	20
17	23
18	26
19	28
20	30
21	32
22	33
23	35
24	37
25	38
26	40
27	41
28	43
29	44
30	46
31	47
32	49
33	50
34	51
35	53
36	54
37	56
38	58
39	59
40	61
41	63
42	64
43	66
44	68
45	70
46	73
47	75
48	78
49	82
50	86
51	92
52	100

BREAST-Q™ - REDUCTION MODULE (POSTOPERATIVE) VERSION 2.0: SATISFACTION WITH NIPPLES

在过去 1 周里，您对以下情况是否感到满意：

	常不满意	常不满意	常不满意	常不满意
a. 乳头在乳房上的位置如何？	**1**	**2**	**3**	**4**
b. 双侧乳头位置是否对称？	**1**	**2**	**3**	**4**
c. 乳头和乳晕的形状如何？	**1**	**2**	**3**	**4**
d. 乳头和乳晕看上去如何？	**1**	**2**	**3**	**4**
e. 乳头的感觉如何？	**1**	**2**	**3**	**4**

BREAST-Q® VERSION 2.0 © Memorial Sloan Kettering Cancer Center and The University of British Columbia, 2017, All rights reserved

---

Instructions: These questions should be considered as stand-alone. Thus, the patient's response is taken as the score form each item. Higher scores reflect a better outcome.

Note to Investigators: This scale can be used independently of the other scales.

The BREAST-Q, authored by Drs. Andrea Pusic, Anne Klassen and Stefan Cano, is the copyright of Memorial Sloan Kettering Cancer Center and The University of British Columbia (Copyright ©2017, Memorial Sloan Kettering Cancer Center and the University of British Columbia). The BREAST-Q has been provided under license from Memorial Sloan Kettering Cancer Center and must not be copied, distributed or used in any way without the prior consent of Memorial Sloan Kettering Cancer Center.

BREAST-Q™ - REDUCTION MODULE (POSTOPERATIVE) VERSION 2.0: SATISFACTION WITH OUTCOME

我们希望知道您对这次手术**效果的**评**价**，请告知您对以下每一个观点**是否**赞同：

	**不同意**	**大抵同意**	**非常同意**
a. 对我来说这次手术是个正确的选择。	**1**	**2**	**3**
b. **我会建**议和我有一样情况的其他女性选择这个手术。	**1**	**2**	**3**
c. **再次**选择，**我**还是愿意进行手术。	**1**	**2**	**3**
d. 总的来说，对整个治疗经过都比较满意。	**1**	**2**	**3**
e. 这个手术使我的生活变得更美好。	**1**	**2**	**3**
f. **做**这个手术我毫不后悔。	**1**	**2**	**3**
g. 手术的效果完全符合我的预期。	**1**	**2**	**3**
h. 手术的结果和我计划的一样。	**1**	**2**	**3**

BREAST-Q® VERSION 2.0 © Memorial Sloan Kettering Cancer Center and The University of British Columbia, 2017, All rights reserved

---

Note to Investigators: This scale can be used independently of the other scales.

The BREAST-Q, authored by Drs. Andrea Pusic, Anne Klassen and Stefan Cano, is the copyright of Memorial Sloan Kettering Cancer Center and The University of British Columbia (Copyright ©2017, Memorial Sloan Kettering Cancer Center and the University of British Columbia). The BREAST-Q has been provided under license from Memorial Sloan Kettering Cancer Center and must not be copied, distributed or used in any way without the prior consent of Memorial Sloan Kettering Cancer Center.

BREAST-Q^{TM} - REDUCTION MODULE (POSTOPERATIVE) VERSION 2.0: SATISFACTION WITH OUTCOME CONVERSION TABLE

Instructions: If missing data is less than 50% of the scale's items, insert the mean of the completed items. Use the Conversion Table below to convert the raw scale summed score into a score from 0 (worst) to 100 (best). Higher scores reflect a better outcome.

SUM SCORE	EQUIVALENT RASCH TRANSFORMED SCORE (0-100)
8	0
9	17
10	25
11	31
12	36
13	39
14	43
15	46
16	49
17	52
18	56
19	59
20	63
21	68
22	76
23	86
24	100

BREAST-Q™ - REDUCTION MODULE (POSTOPERATIVE) VERSION 2.0: PATIENT EXPERIENCE: SATISFACTION WITH INFORMATION

对于从整形外科医生那里获得的以下信息，您是否感觉满意：

	非常不满意	比较不满意	比较满意	非常满意
a. 手术如何进行。	1	2	3	4
b. 可能出现的并发症。	1	2	3	4
c. 治疗及恢复的时间。	1	2	3	4
d. 如何选择合适的乳房大小。	1	2	3	4
e. 可能造成乳头敏感度的降低。	1	2	3	4
f. 手术后乳房可能的大小。	1	2	3	4
g. 可能造成乳头及乳晕区域血供的缺失。	1	2	3	4
h. 手术后的伤口护理方法。	1	2	3	4
i. 手术后乳房可能的大小。	1	2	3	4
j. 瘢痕的外观。	1	2	3	4
k. 手术可能对以后的乳房肿瘤检查产生干扰（如乳腺 X 线检查，自我检查等）。	1	2	3	4
l. 瘢痕治疗的方法。	1	2	3	4
m. 手术可能会对哺乳造成影响。（非必答）	1	2	3	4

BREAST-Q® VERSION 2.0 © Memorial Sloan Kettering Cancer Center and The University of British Columbia, 2017, All rights reserved

Note to Investigators: This scale can be used independently of the other scales. Depending on the use of this scale, you may wish to add the following statement to the stem for clarity. 'These questions ask about the surgeon who performed your most recent surgery.'

The BREAST-Q, authored by Drs. Andrea Pusic, Anne Klassen and Stefan Cano, is the copyright of Memorial Sloan Kettering Cancer Center and The University of British Columbia (Copyright ©2017, Memorial Sloan Kettering Cancer Center and the University of British Columbia). The BREAST-Q has been provided under license from Memorial Sloan Kettering Cancer Center and must not be copied, distributed or used in any way without the prior consent of Memorial Sloan Kettering Cancer Center.

BREAST-Q^{TM} - REDUCTION MODULE (POSTOPERATIVE) VERSION 2.0: PATIENT EXPERIENCE: SATISFACTION WITH INFORMATION CONVERSION TABLE

Instructions: If missing data is less than 50% of the scale's items, insert the mean of the completed items. Use the Conversion Table below to convert the raw scale summed score into a score from 0 (worst) to 100 (best). Higher scores reflect a better outcome.

SUM SCORE	EQUIVALENT RASCH TRANSFORMED SCORE (0-100)
13	0
14	13
15	19
16	23
17	26
18	29
19	31
20	33
21	34
22	36
23	37
24	39
25	40
26	41
27	42
28	44
29	45
30	46
31	47
32	48
33	50
34	51
35	52
36	53
37	55
38	56
39	57
40	59
41	60
42	62
43	64
44	66
45	68
46	70
47	72
48	75
49	79
50	84
51	90
52	100

BREAST-Q™ - REDUCTION MODULE (POSTOPERATIVE) VERSION 2.0: PATIENT EXPERIENCE: SATISFACTION WITH SURGEON

以下问题与您的整形医生有关。您觉得他/她：

	非常不满意	比较不满意	比较满意	非常满意
a. 称职吗？	1	2	3	4
b. 给予了您信心吗？	1	2	3	4
c. 让您参与了方案的制定吗？	1	2	3	4
d. 让人放心吗？	1	2	3	4
e. 回答了您所有的问题吗？	1	2	3	4
f. 让您觉得舒适吗？	1	2	3	4
g. 细心周全吗？	1	2	3	4
h. 容易交流吗？	1	2	3	4
i. 能理解您的想法吗？	1	2	3	4
j. 能察觉您的想法吗？	1	2	3	4
k. 为您安排了诉说顾虑的时间吗？	1	2	3	4
l. 在您有顾虑时听您诉说了吗？	1	2	3	4

BREAST-Q® VERSION 2.0 © Memorial Sloan Kettering Cancer Center and The University of British Columbia, 2017, All rights reserved

Note to Investigators: This scale can be used independently of the other scales. This scale is exactly the same across all BREAST-Q Postoperative Modules. Depending on the use of this scale, you may wish to add the following statement to the stem for clarity. 'These questions ask about the surgeon who performed your most recent surgery.'

The BREAST-Q, authored by Drs. Andrea Pusic, Anne Klassen and Stefan Cano, is the copyright of Memorial Sloan Kettering Cancer Center and The University of British Columbia (Copyright ©2017, Memorial Sloan Kettering Cancer Center and the University of British Columbia). The BREAST-Q has been provided under license from Memorial Sloan Kettering Cancer Center and must not be copied, distributed or used in any way without the prior consent of Memorial Sloan Kettering Cancer Center.

BREAST-Q^{TM} - REDUCTION MODULE (POSTOPERATIVE) VERSION 2.0: PATIENT EXPERIENCE: SATISFACTION WITH SURGEON CONVERSION TABLE

Instructions: If missing data is less than 50% of the scale's items, insert the mean of the completed items. Use the Conversion Table below to convert the raw scale summed score into a score from 0 (worst) to 100 (best). Higher scores reflect a better outcome.

SUM SCORE	EQUIVALENT RASCH TRANSFORMED SCORE (0-100)
12	0
13	16
14	21
15	24
16	26
17	29
18	30
19	32
20	34
21	35
22	36
23	38
24	39
25	40
26	42
27	43
28	44
29	46
30	47
31	49
32	50
33	52
34	54
35	56
36	58
37	60
38	62
39	64
40	67
41	69
42	72
43	75
44	78
45	81
46	86
47	92
48	100

BREAST-Q™ - REDUCTION MODULE (POSTOPERATIVE) VERSION 2.0: PATIENT EXPERIENCE: SATISFACTION WITH MEDICAL TEAM

以下是有关医疗团队其他成员（**如**：手术前后负责照顾您的护士和其他医生）**的**问题。**您**觉得他们：

	完全不同意	比较不同意	比较同意	完全同意
a. 专业吗？	1	2	3	4
b. 对您尊重吗？	1	2	3	4
c. 专业知识充足吗？	1	2	3	4
d. 友善吗？	1	2	3	4
e. 让您感到舒适吗？	1	2	3	4
f. 细心周全吗？	1	2	3	4
g. 为您安排了诉说顾虑的时间吗？	1	2	3	4

BREAST-Q® VERSION 2.0 © Memorial Sloan Kettering Cancer Center and The University of British Columbia, 2017, All rights reserved

---

Note to Investigators: This scale can be used independently of the other scales. This scale is exactly the same across all BREAST-Q Postoperative Modules. Depending on the use of this scale, you may modify the stem wording to fit your clinical environment (e.g. medical team may include nurses, physician assistants, or other licensed independent practitioners).

The BREAST-Q, authored by Drs. Andrea Pusic, Anne Klassen and Stefan Cano, is the copyright of Memorial Sloan Kettering Cancer Center and The University of British Columbia (Copyright ©2017, Memorial Sloan Kettering Cancer Center and the University of British Columbia). The BREAST-Q has been provided under license from Memorial Sloan Kettering Cancer Center and must not be copied, distributed or used in any way without the prior consent of Memorial Sloan Kettering Cancer Center.

BREAST-Q™ - REDUCTION MODULE (POSTOPERATIVE) VERSION 2.0: PATIENT EXPERIENCE: SATISFACTION WITH MEDICAL TEAM CONVERSION TABLE

Instructions: If missing data is less than 50% of the scale's items, insert the mean of the completed items. Use the Conversion Table below to convert the raw scale summed score into a score from 0 (worst) to 100 (best). Higher scores reflect a better outcome.

SUM SCORE	EQUIVALENT RASCH TRANSFORMED SCORE (0-100)
7	0
8	0
9	11
10	20
11	27
12	32
13	36
14	40
15	43
16	46
17	50
18	53
19	57
20	61
21	65
22	69
23	73
24	77
25	82
26	86
27	92
28	100

BREAST-Q™ - REDUCTION MODULE (POSTOPERATIVE) VERSION 2.0: PATIENT EXPERIENCE: SATISFACTION WITH OFFICE STAFF

以下问题与**其他工作人员**（**如**：**秘**书、办公室或门诊护士）**有关**。**您**觉得他们：

	**完全不同意**	**比较不同意**	**比较同意**	**完全同意**
a. 专业吗？	**1**	**2**	**3**	**4**
b. 对您尊重吗？	**1**	**2**	**3**	**4**
c. 专业知识充足吗？	**1**	**2**	**3**	**4**
d. **友善**吗？	**1**	**2**	**3**	**4**
e. 让您感到舒适吗？	**1**	**2**	**3**	**4**
f. 细心周全吗？	**1**	**2**	**3**	**4**
g. 为您安排了诉说顾虑的时间吗？	**1**	**2**	**3**	**4**

BREAST-Q® VERSION 2.0 © Memorial Sloan Kettering Cancer Center and The University of British Columbia, 2017, All rights reserved

---

Note to Investigators: This scale can be used independently of the other scales. This scale is exactly the same across all BREAST-Q Postoperative Modules. Depending on the use of this scale, you may modify the stem wording to fit your office environment (e.g. office or clinic nurse).

The BREAST-Q, authored by Drs. Andrea Pusic, Anne Klassen and Stefan Cano, is the copyright of Memorial Sloan Kettering Cancer Center and The University of British Columbia (Copyright ©2017, Memorial Sloan Kettering Cancer Center and the University of British Columbia). The BREAST-Q has been provided under license from Memorial Sloan Kettering Cancer Center and must not be copied, distributed or used in any way without the prior consent of Memorial Sloan Kettering Cancer Center.

BREAST-Q™ - REDUCTION MODULE (POSTOPERATIVE) VERSION 2.0: PATIENT EXPERIENCE: SATISFACTION WITH OFFICE STAFF CONVERSION TABLE

Instructions: If missing data is less than 50% of the scale's items, insert the mean of the completed items. Use the Conversion Table below to convert the raw scale summed score into a score from 0 (worst) to 100 (best). Higher scores reflect a better outcome.

SUM SCORE	EQUIVALENT RASCH TRANSFORMED SCORE (0-100)
7	0
8	17
9	24
10	28
11	31
12	34
13	37
14	39
15	41
16	44
17	46
18	49
19	51
20	54
21	58
22	61
23	65
24	70
25	75
26	81
27	89
28	100
