# Supplementary material for: Clinical study on single-port endoscopic resection via a gasless transaxillary approach in the treatment of breast fibroadenoma in adolescents
Source: BMC Surg. 2023 Sep 14;23:279. doi: 10.1186/s12893-023-02186-1 (PMC10503113; doi:10.1186/s12893-023-02186-1)
Supplement: Supplementary file 6 — Supplementary Material 6 [file 12893_2023_2186_MOESM6_ESM.pdf]

BREAST-Q Version 2.0©

Reduction/Mastopexy Module
Pre- and Postoperative Scales

English Version

The BREAST-Q, authored by Drs. Andrea Pusic, Anne Klassen and Stefan Cano, is the copyright of Memorial Sloan Kettering Cancer Center and The University of British Columbia (Copyright ©2017, Memorial Sloan Kettering Cancer Center and the University of British Columbia). The BREAST-Q has been provided under license from Memorial Sloan Kettering Cancer Center and must not be copied, distributed or used in any way without the prior consent of Memorial Sloan Kettering Cancer Center.

NOTE TO LICENSED USERS

Each scale in this booklet can be used independently of the other scales (i.e., you don't have to use them all). For each scale that you use, the patients or research participants DO NOT NEED TO SEE:

- title of each scale
- notes at the bottom of the scale
- scoring table for the scale

We are able to provide you with a Word version of this booklet if needed. Send an email to: qportfolioteam@gmail.com

Patients or research participants only need to see the instructions, items, response options and the copyright notice at the bottom of the scale. Here's an example:

Thinking of your sexuality, how often do you generally feel:

	None of the time	A little of the time	Some of the time	Most of the time	All of the time
a. Comfortable/at ease during sexual activity?	1	2	3	4	5
b. Confident sexually?	1	2	3	4	5
c. Satisfied with your sex life?	1	2	3	4	5
d. Sexually attractive in your clothes?	1	2	3	4	5
e. Sexy when unclothed?	1	2	3	4	5

BREAST-Q VERSION 2.0 © 2017 Memorial Sloan Kettering Cancer Center and The University of British Columbia. All rights reserved.

AS A REMINDER: In the license you signed, you agreed to the following:

1. You will not change this questionnaire in any way
2. You will not translate this questionnaire without permission
3. You will not give this questionnaire to an unlicensed user
4. You will not reproduce this questionnaire in publications or other materials

BREAST-Q™ - REDUCTION MODULE (PRE- AND POSTOPERATIVE) VERSION 2.0: PSYCHOSOCIAL WELL-BEING

With your breasts in mind, in the past week, how often have you felt:

	None of the time	A little of the time	Some of the time	Most of the time	All of the time
a. Confident in a social setting?	**1**	**2**	**3**	**4**	**5**
b. Of equal worth to other women?	**1**	**2**	**3**	**4**	**5**
c. Good about yourself?	**1**	**2**	**3**	**4**	**5**
d. Self-assured?	**1**	**2**	**3**	**4**	**5**
e. Confident in your clothes?	**1**	**2**	**3**	**4**	**5**
f. Accepting of your body?	**1**	**2**	**3**	**4**	**5**
g. That your appearance matches who you are inside?	**1**	**2**	**3**	**4**	**5**
h. Confident about your body?	**1**	**2**	**3**	**4**	**5**
i. Attractive?	**1**	**2**	**3**	**4**	**5**

BREAST-Q VERSION 2.0 © 2017 Memorial Sloan Kettering Cancer Center and The University of British Columbia. All rights reserved

---

Note to Investigators: This scale can be used independently of the other scales.

The BREAST-Q, authored by Drs. Andrea Pusic, Anne Klassen and Stefan Cano, is the copyright of Memorial Sloan Kettering Cancer Center and The University of British Columbia (Copyright ©2017, Memorial Sloan Kettering Cancer Center and the University of British Columbia). The BREAST-Q has been provided under license from Memorial Sloan Kettering Cancer Center and must not be copied, distributed or used in any way without the prior consent of Memorial Sloan Kettering Cancer Center.

BREAST-Q™ - REDUCTION MODULE (PRE- AND POSTOPERATIVE) VERSION 2.0: PSYCHOSOCIAL WELL-BEING CONVERSION TABLE

Instructions: If missing data is less than 50% of the scale's items, insert the mean of the completed items. Use the Conversion Table below to convert the raw scale summed score into a score from 0 (worst) to 100 (best). Higher scores reflect a better outcome.

SUM SCORE	EQUIVALENT RASCH TRANSFORMED SCORE (0-100)
9	0
10	14
11	18
12	21
13	24
14	26
15	28
16	30
17	32
18	33
19	35
20	36
21	38
22	39
23	41
24	42
25	44
26	45
27	47
28	49
29	50
30	52
31	54
32	56
33	59
34	61
35	64
36	66
37	69
38	72
39	75
40	78
41	81
42	84
43	88
44	93
45	100

BREAST-Q™ - REDUCTION MODULE (PRE- AND POSTOPERATIVE) VERSION 2.0: SEXUAL WELL-BEING

Thinking of your sexuality, how often do you generally feel:

	None of the time	A little of the time	Some of the time	Most of the time	All of the time
a. Comfortable/at ease during sexual activity?	**1**	**2**	**3**	**4**	**5**
b. Confident sexually?	**1**	**2**	**3**	**4**	**5**
c. Satisfied with your sex life?	**1**	**2**	**3**	**4**	**5**
d. Sexually attractive in your clothes?	**1**	**2**	**3**	**4**	**5**
e. Sexy when unclothed?	**1**	**2**	**3**	**4**	**5**

BREAST-Q VERSION 2.0 © 2017 Memorial Sloan Kettering Cancer Center and The University of British Columbia. All rights reserved.

Note to Investigators: This scale can be used independently of the other scales. The following statement can be added to the stem to provide an opportunity for the patient to decline completing this scale. 'The following questions ask about your sexual well-being. If you are uncomfortable answering these questions or do not feel that they apply to you, please check the box and skip the questions that follow.'

The BREAST-Q, authored by Drs. Andrea Pusic, Anne Klassen and Stefan Cano, is the copyright of Memorial Sloan Kettering Cancer Center and The University of British Columbia (Copyright ©2017, Memorial Sloan Kettering Cancer Center and the University of British Columbia). The BREAST-Q has been provided under license from Memorial Sloan Kettering Cancer Center and must not be copied, distributed or used in any way without the prior consent of Memorial Sloan Kettering Cancer Center.

BREAST-Q™ - REDUCTION MODULE (PRE- AND POSTOPERATIVE) VERSION 2.0: SEXUAL WELL-BEING CONVERSION TABLE

Instructions: If missing data is less than 50% of the scale's items, insert the mean of the completed items. Use the Conversion Table below to convert the raw scale summed score into a score from 0 (worst) to 100 (best). Higher scores reflect a better outcome.

SUM SCORE	EQUIVALENT RASCH TRANSFORMED SCORE (0-100)
5	0
6	18
7	23
8	28
9	31
10	34
11	37
12	39
13	42
14	44
15	47
16	50
17	53
18	56
19	60
20	65
21	71
22	76
23	82
24	90
25	100

BREAST-Q™ - REDUCTION MODULE (PRE- AND POSTOPERATIVE) VERSION 2.0: PHYSICAL WELL-BEING

In the past week, how often have you experienced:

	None of the time	Some of the time	All of the time
a. Headaches?	**1**	**2**	**3**
b. Pain in your breast area?	**1**	**2**	**3**
c. Lack of energy?	**1**	**2**	**3**
d. Difficulty doing vigorous physical activities (e.g. running or exercising)?	**1**	**2**	**3**
e. Feeling physically unbalanced?	**1**	**2**	**3**
f. Shoulder pain?	**1**	**2**	**3**
g. Difficulty sleeping because of discomfort in your breast area?	**1**	**2**	**3**
h. Neck pain?	**1**	**2**	**3**
i. Painful gouges or grooves in your shoulders from your bra straps?	**1**	**2**	**3**
j. Feeling physically uncomfortable?	**1**	**2**	**3**
k. Rashes under your breasts?	**1**	**2**	**3**
l. Back pain?	**1**	**2**	**3**
m. Arm pain?	**1**	**2**	**3**
n. Pain, numbness or tingling in your hands because of your breast size?	**1**	**2**	**3**

BREAST-Q VERSION 2.0 © 2017 Memorial Sloan Kettering Cancer Center and The University of British Columbia. All rights reserved

---

Note to Investigators: This scale can be used independently of the other scales.

The BREAST-Q, authored by Drs. Andrea Pusic, Anne Klassen and Stefan Cano, is the copyright of Memorial Sloan Kettering Cancer Center and The University of British Columbia (Copyright ©2017, Memorial Sloan Kettering Cancer Center and the University of British Columbia). The BREAST-Q has been provided under license from Memorial Sloan Kettering Cancer Center and must not be copied, distributed or used in any way without the prior consent of Memorial Sloan Kettering Cancer Center.

BREAST-Q™ - REDUCTION MODULE (PRE- AND POSTOPERATIVE) VERSION 2.0: PHYSICAL WELL-BEING CONVERSION TABLE

Instructions: Items 'a' and 'b' are stand-alone items that are not included in the scale score. Recode items c, d, e, f, g, h, i, j, k, l, m, and n as follows: "None of the time" = 3; "Some of the time" = 2; "All of the time" = 1. If missing data is less than 50% of the scale's items, insert the mean of the completed items. Use the Conversion Table below to convert the raw scale summed score into a score from 0 (worst) to 100 (best). Higher scores reflect a better outcome.

SUM SCORE	EQUIVALENT RASCH TRANSFORMED SCORE (0-100)
12	0
13	14
14	20
15	25
16	28
17	31
18	34
19	37
20	40
21	42
22	44
23	47
24	49
25	51
26	54
27	56
28	59
29	62
30	65
31	68
32	72
33	77
34	82
35	90
36	100

BREAST-Q™ - REDUCTION MODULE (PREOPERATIVE) VERSION 2.0: SATISFACTION WITH BREASTS

With your breasts in mind, in the past week, how satisfied or dissatisfied have you been with:

	Very Dissatisfied	Somewhat Dissatisfied	Somewhat Satisfied	Very Satisfied
a. How your breasts look in clothes?	**1**	**2**	**3**	**4**
b. How your breast size matches the rest of your body?	**1**	**2**	**3**	**4**
c. The size of your breasts?	**1**	**2**	**3**	**4**
d. The shape of your breasts when you are wearing a bra?	**1**	**2**	**3**	**4**
e. How equal in size your breasts are to each other?	**1**	**2**	**3**	**4**
f. How comfortably your bras fit?	**1**	**2**	**3**	**4**
g. The shape of your breasts when you are not wearing a bra?	**1**	**2**	**3**	**4**
h. How you look in the mirror clothed?	**1**	**2**	**3**	**4**
i. How your breasts sit/hang on your chest?	**1**	**2**	**3**	**4**
j. How normal your breasts look?	**1**	**2**	**3**	**4**
k. How you look in the mirror unclothed?	**1**	**2**	**3**	**4**

BREAST-Q VERSION 2.0 © 2017 Memorial Sloan Kettering Cancer Center and The University of British Columbia. All rights reserved

---

Note to Investigators: This scale can be used independently of the other scales.

The BREAST-Q, authored by Drs. Andrea Pusic, Anne Klassen and Stefan Cano, is the copyright of Memorial Sloan Kettering Cancer Center and The University of British Columbia (Copyright ©2017, Memorial Sloan Kettering Cancer Center and the University of British Columbia). The BREAST-Q has been provided under license from Memorial Sloan Kettering Cancer Center and must not be copied, distributed or used in any way without the prior consent of Memorial Sloan Kettering Cancer Center.

BREAST-Q™ - REDUCTION MODULE (PREOPERATIVE) VERSION 2.0: SATISFACTION WITH BREASTS CONVERSION TABLE

Instructions: If missing data is less than 50% of the scale's items, insert the mean of the completed items. Use the Conversion Table below to convert the raw scale summed score into a score from 0 (worst) to 100 (best). Higher scores reflect a better outcome.

SUM SCORE	EQUIVALENT RASCH TRANSFORMED SCORE (0-100)
11	0
12	11
13	17
14	21
15	24
16	26
17	29
18	31
19	33
20	35
21	36
22	38
23	40
24	41
25	43
26	45
27	46
28	48
29	50
30	52
31	53
32	55
33	57
34	59
35	61
36	63
37	66
38	68
39	71
40	74
41	78
42	82
43	89
44	100

BREAST-Q™ - REDUCTION MODULE (POSTOPERATIVE) VERSION 2.0: SATISFACTION WITH BREASTS

With your breasts in mind, in the past week, how satisfied or dissatisfied have you been with:

	Very Dissatisfied	Somewhat Dissatisfied	Somewhat Satisfied	Very Satisfied
a. How your breasts look in clothes?	**1**	**2**	**3**	**4**
b. How your breast size matches the rest of your body?	**1**	**2**	**3**	**4**
c. The size of your breasts?	**1**	**2**	**3**	**4**
d. The shape of your breasts when you are wearing a bra?	**1**	**2**	**3**	**4**
e. How equal in size your breasts are to each other?	**1**	**2**	**3**	**4**
f. How comfortably your bras fit?	**1**	**2**	**3**	**4**
g. The shape of your breasts when you are not wearing a bra?	**1**	**2**	**3**	**4**
h. How you look in the mirror clothed?	**1**	**2**	**3**	**4**
i. How your breasts sit/hang on your chest?	**1**	**2**	**3**	**4**
j. How normal your breasts look?	**1**	**2**	**3**	**4**
k. The location of your scars?	**1**	**2**	**3**	**4**
l. How your scars look?	**1**	**2**	**3**	**4**
m. How you look in the mirror unclothed?	**1**	**2**	**3**	**4**

BREAST-Q VERSION 2.0 © 2017 Memorial Sloan Kettering Cancer Center and The University of British Columbia. All rights reserved

---

Note to Investigators: This scale can be used independently of the other scales.

The BREAST-Q, authored by Drs. Andrea Pusic, Anne Klassen and Stefan Cano, is the copyright of Memorial Sloan Kettering Cancer Center and The University of British Columbia (Copyright ©2017, Memorial Sloan Kettering Cancer Center and the University of British Columbia). The BREAST-Q has been provided under license from Memorial Sloan Kettering Cancer Center and must not be copied, distributed or used in any way without the prior consent of Memorial Sloan Kettering Cancer Center.

BREAST-Q™ - REDUCTION MODULE (POSTOPERATIVE) VERSION 2.0: SATISFACTION WITH BREASTS CONVERSION TABLE

Instructions: If missing data is less than 50% of the scale's items, insert the mean of the completed items. Use the Conversion Table below to convert the raw scale summed score into a score from 0 (worst) to 100 (best). Higher scores reflect a better outcome.

SUM SCORE	EQUIVALENT RASCH TRANSFORMED SCORE (0-100)
13	0
14	11
15	16
16	20
17	23
18	26
19	28
20	30
21	32
22	33
23	35
24	37
25	38
26	40
27	41
28	43
29	44
30	46
31	47
32	49
33	50
34	51
35	53
36	54
37	56
38	58
39	59
40	61
41	63
42	64
43	66
44	68
45	70
46	73
47	75
48	78
49	82
50	86
51	92
52	100

BREAST-Q™ - REDUCTION MODULE (POSTOPERATIVE) VERSION 2.0: SATISFACTION WITH NIPPLES

In the past week, how satisfied or dissatisfied have you been with:

	Very Dissatisfied	Somewhat Dissatisfied	Somewhat Satisfied	Very Satisfied
a. How high or low your nipples are on your breasts?	**1**	**2**	**3**	**4**
b. How your nipples are lined up in relation to each other?	**1**	**2**	**3**	**4**
c. The shape of your nipples and areolas?	**1**	**2**	**3**	**4**
d. How your nipples and areolas look?	**1**	**2**	**3**	**4**
e. The amount of sensation (feeling) in your nipples?	**1**	**2**	**3**	**4**

BREAST-Q VERSION 2.0 © 2017 Memorial Sloan Kettering Cancer Center and The University of British Columbia. All rights reserved

---

Instructions: These questions should be considered as stand-alone. Thus, the patient's response is taken as the score for each item. Higher scores reflect a better outcome.

Note to Investigators: This scale can be used independently of the other scales.

The BREAST-Q, authored by Drs. Andrea Pusic, Anne Klassen and Stefan Cano, is the copyright of Memorial Sloan Kettering Cancer Center and The University of British Columbia (Copyright ©2017, Memorial Sloan Kettering Cancer Center and the University of British Columbia). The BREAST-Q has been provided under license from Memorial Sloan Kettering Cancer Center and must not be copied, distributed or used in any way without the prior consent of Memorial Sloan Kettering Cancer Center.

BREAST-Q^{TM} - REDUCTION MODULE (POSTOPERATIVE) VERSION 2.0: SATISFACTION WITH OUTCOME

We would like to know how you feel about the outcome of your breast surgery. Please indicate how much you agree or disagree with each statement:

	Disagree	Somewhat Agree	Definitely Agree
a. Having surgery was the right decision for me.	**1**	**2**	**3**
b. I would encourage other women in my situation to have breast reduction surgery.	**1**	**2**	**3**
c. I would do it again.	**1**	**2**	**3**
d. Overall the surgery was a positive experience.	**1**	**2**	**3**
e. Having surgery changed my life for the better.	**1**	**2**	**3**
f. I have no regrets about having surgery.	**1**	**2**	**3**
g. The outcome perfectly matched my expectations.	**1**	**2**	**3**
h. It turned out exactly as I had planned.	**1**	**2**	**3**

BREAST-Q VERSION 2.0 © 2017 Memorial Sloan Kettering Cancer Center and The University of British Columbia. All rights reserved

---

Note to Investigators: This scale can be used independently of the other scales.

The BREAST-Q, authored by Drs. Andrea Pusic, Anne Klassen and Stefan Cano, is the copyright of Memorial Sloan Kettering Cancer Center and The University of British Columbia (Copyright ©2017, Memorial Sloan Kettering Cancer Center and the University of British Columbia). The BREAST-Q has been provided under license from Memorial Sloan Kettering Cancer Center and must not be copied, distributed or used in any way without the prior consent of Memorial Sloan Kettering Cancer Center.

BREAST-Q™ - REDUCTION MODULE (POSTOPERATIVE) VERSION 2.0: SATISFACTION WITH OUTCOME CONVERSION TABLE

Instructions: If missing data is less than 50% of the scale's items, insert the mean of the completed items. Use the Conversion Table below to convert the raw scale summed score into a score from 0 (worst) to 100 (best). Higher scores reflect a better outcome.

SUM SCORE	EQUIVALENT RASCH TRANSFORMED SCORE (0-100)
8	0
9	17
10	25
11	31
12	36
13	39
14	43
15	46
16	49
17	52
18	56
19	59
20	63
21	68
22	76
23	86
24	100

BREAST-Q^{TM} - REDUCTION MODULE (POSTOPERATIVE) VERSION 2.0: PATIENT EXPERIENCE: SATISFACTION WITH INFORMATION

How satisfied or dissatisfied were you with the information you received from your plastic surgeon about:

	Very Dissatisfied	Somewhat Dissatisfied	Somewhat Satisfied	Very Satisfied
a. How the surgery was to be done?	1	2	3	4
b. Possible complications?	1	2	3	4
c. Healing and recovery time?	1	2	3	4
d. How to choose a breast size that would suit what you wanted?	1	2	3	4
e. The potential for loss of sensation in your nipples?	1	2	3	4
f. What size you could expect your breasts to be after surgery?	1	2	3	4
g. Potential for loss of blood supply to your nipple area?	1	2	3	4
h. How to care for your incisions after surgery?	1	2	3	4
i. What you could expect your breasts to look like after surgery?	1	2	3	4
j. What the scars would look like?	1	2	3	4
k. How the surgery could affect future breast cancer screening (e.g. mammogram, self-examinations)?	1	2	3	4
l. Options to help with scarring?	1	2	3	4
m. How the surgery could affect breast-feeding? (only answer if applicable)	1	2	3	4

BREAST-Q VERSION 2.0 © 2017 Memorial Sloan Kettering Cancer Center and The University of British Columbia. All rights reserved

---

Note to Investigators: This scale can be used independently of the other scales. Depending on the use of this scale, you may wish to add the following statement to the stem for clarity. 'These questions ask about the surgeon who performed your most recent surgery.'

The BREAST-Q, authored by Drs. Andrea Pusic, Anne Klassen and Stefan Cano, is the copyright of Memorial Sloan Kettering Cancer Center and The University of British Columbia (Copyright ©2017, Memorial Sloan Kettering Cancer Center and the University of British Columbia). The BREAST-Q has been provided under license from Memorial Sloan Kettering Cancer Center and must not be copied, distributed or used in any way without the prior consent of Memorial Sloan Kettering Cancer Center.

BREAST-Q™ - REDUCTION MODULE (POSTOPERATIVE) VERSION 2.0:
PATIENT EXPERIENCE: SATISFACTION WITH INFORMATION CONVERSION TABLE

Instructions: If missing data is less than 50% of the scale's items, insert the mean of the completed items. Use the Conversion Table below to convert the raw scale summed score into a score from 0 (worst) to 100 (best). Higher scores reflect a better outcome.

SUM SCORE	EQUIVALENT RASCH TRANSFORMED SCORE (0-100)
13	0
14	13
15	19
16	23
17	26
18	29
19	31
20	33
21	34
22	36
23	37
24	39
25	40
26	41
27	42
28	44
29	45
30	46
31	47
32	48
33	50
34	51
35	52
36	53
37	55
38	56
39	57
40	59
41	60
42	62
43	64
44	66
45	68
46	70
47	72
48	75
49	79
50	84
51	90
52	100

BREAST-Q™ - REDUCTION MODULE (POSTOPERATIVE) VERSION 2.0: PATIENT EXPERIENCE: SATISFACTION WITH SURGEON

These questions ask about your plastic surgeon. Did you feel that he/she:

	Definitely Disagree	Somewhat Disagree	Somewhat Agree	Definitely Agree
a. Was professional?	1	2	3	4
b. Gave you confidence?	1	2	3	4
c. Involved you in the decision-making process?	1	2	3	4
d. Was reassuring?	1	2	3	4
e. Answered all your questions?	1	2	3	4
f. Made you feel comfortable?	1	2	3	4
g. Was thorough?	1	2	3	4
h. Was easy to talk to?	1	2	3	4
i. Understood what you wanted?	1	2	3	4
j. Was sensitive?	1	2	3	4
k. Made time for your concerns?	1	2	3	4
l. Was available when you had concerns?	1	2	3	4

BREAST-Q VERSION 2.0 © 2017 Memorial Sloan Kettering Cancer Center and The University of British Columbia. All rights reserved

---

Note to Investigators: This scale can be used independently of the other scales. This scale is exactly the same across all BREAST-Q Postoperative Modules. Depending on the use of this scale, you may wish to add the following statement to the stem for clarity. 'These questions ask about the surgeon who performed your most recent surgery.'

The BREAST-Q, authored by Drs. Andrea Pusic, Anne Klassen and Stefan Cano, is the copyright of Memorial Sloan Kettering Cancer Center and The University of British Columbia (Copyright ©2017, Memorial Sloan Kettering Cancer Center and the University of British Columbia). The BREAST-Q has been provided under license from Memorial Sloan Kettering Cancer Center and must not be copied, distributed or used in any way without the prior consent of Memorial Sloan Kettering Cancer Center.

BREAST-Q^{TM} - REDUCTION MODULE (POSTOPERATIVE) VERSION 2.0:
PATIENT EXPERIENCE: SATISFACTION WITH SURGEON CONVERSION TABLE

Instructions: If missing data is less than 50% of the scale's items, insert the mean of the completed items. Use the Conversion Table below to convert the raw scale summed score into a score from 0 (worst) to 100 (best). Higher scores reflect a better outcome.

SUM SCORE	EQUIVALENT RASCH TRANSFORMED SCORE (0-100)
12	0
13	16
14	21
15	24
16	26
17	29
18	30
19	32
20	34
21	35
22	36
23	38
24	39
25	40
26	42
27	43
28	44
29	46
30	47
31	49
32	50
33	52
34	54
35	56
36	58
37	60
38	62
39	64
40	67
41	69
42	72
43	75
44	78
45	81
46	86
47	92
48	100

BREAST-Q™ - REDUCTION MODULE (POSTOPERATIVE) VERSION 2.0: PATIENT EXPERIENCE: SATISFACTION WITH MEDICAL TEAM

These questions ask about members of the medical team other than the surgeon. Did you feel that they:

	Definitely Disagree	Somewhat Disagree	Somewhat Agree	Definitely Agree
a. Were professional?	1	2	3	4
b. Treated you with respect?	1	2	3	4
c. Were knowledgeable?	1	2	3	4
d. Were friendly and kind?	1	2	3	4
e. Made you feel comfortable?	1	2	3	4
f. Were thorough?	1	2	3	4
g. Made time for your concerns?	1	2	3	4

BREAST-Q VERSION 2.0 © 2017 Memorial Sloan Kettering Cancer Center and The University of British Columbia. All rights reserved

Note to Investigators: This scale can be used independently of the other scales. This scale is exactly the same across all BREAST-Q Postoperative Modules. Depending on the use of this scale, you may modify the stem wording to fit your clinical environment. (e.g. medical team may include nurses, physician assistants, or other licensed independent practitioners)

The BREAST-Q, authored by Drs. Andrea Pusic, Anne Klassen and Stefan Cano, is the copyright of Memorial Sloan Kettering Cancer Center and The University of British Columbia (Copyright ©2017, Memorial Sloan Kettering Cancer Center and the University of British Columbia). The BREAST-Q has been provided under license from Memorial Sloan Kettering Cancer Center and must not be copied, distributed or used in any way without the prior consent of Memorial Sloan Kettering Cancer Center.

BREAST-Q™ - REDUCTION MODULE (POSTOPERATIVE) VERSION 2.0:
PATIENT EXPERIENCE: SATISFACTION WITH MEDICAL TEAM CONVERSION TABLE

Instructions: If missing data is less than 50% of the scale's items, insert the mean of the completed items. Use the Conversion Table below to convert the raw scale summed score into a score from 0 (worst) to 100 (best). Higher scores reflect a better outcome.

SUM SCORE	EQUIVALENT RASCH TRANSFORMED SCORE (0-100)
7	0
8	0
9	11
10	20
11	27
12	32
13	36
14	40
15	43
16	46
17	50
18	53
19	57
20	61
21	65
22	69
23	73
24	77
25	82
26	86
27	92
28	100

BREAST-Q™ - REDUCTION MODULE (POSTOPERATIVE) VERSION 2.0:
PATIENT EXPERIENCE: SATISFACTION WITH OFFICE STAFF

These questions ask about members of the office staff (e.g. secretaries). Did you feel that they:

	**Definitely Disagree**	**Somewhat Disagree**	**Somewhat Agree**	**Definitely Agree**
a. Were professional?	**1**	**2**	**3**	**4**
b. Treated you with respect?	**1**	**2**	**3**	**4**
c. Were knowledgeable?	**1**	**2**	**3**	**4**
d. Were friendly and kind?	**1**	**2**	**3**	**4**
e. Made you feel comfortable?	**1**	**2**	**3**	**4**
f. Were thorough?	**1**	**2**	**3**	**4**
g. Made time for your concerns?	**1**	**2**	**3**	**4**

BREAST-Q VERSION 2.0 © 2017 Memorial Sloan Kettering Cancer Center and The University of British Columbia. All rights reserved

---

Note to Investigators: This scale can be used independently of the other scales. This scale is exactly the same across all BREAST-Q Postoperative Modules. Depending on the use of this scale, you may modify the stem wording to fit your office environment. (e.g. office or clinic nurse)

The BREAST-Q, authored by Drs. Andrea Pusic, Anne Klassen and Stefan Cano, is the copyright of Memorial Sloan Kettering Cancer Center and The University of British Columbia (Copyright ©2017, Memorial Sloan Kettering Cancer Center and the University of British Columbia). The BREAST-Q has been provided under license from Memorial Sloan Kettering Cancer Center and must not be copied, distributed or used in any way without the prior consent of Memorial Sloan Kettering Cancer Center.

BREAST-Q^{TM} - REDUCTION MODULE (POSTOPERATIVE) VERSION 2.0: PATIENT EXPERIENCE: SATISFACTION WITH OFFICE STAFF CONVERSION TABLE

Instructions: If missing data is less than 50% of the scale's items, insert the mean of the completed items. Use the Conversion Table below to convert the raw scale summed score into a score from 0 (worst) to 100 (best). Higher scores reflect a better outcome.

SUM SCORE	EQUIVALENT RASCH TRANSFORMED SCORE (0-100)
7	0
8	17
9	24
10	28
11	31
12	34
13	37
14	39
15	41
16	44
17	46
18	49
19	51
20	54
21	58
22	61
23	65
24	70
25	75
26	81
27	89
28	100
