# Supplementary material for: Clinical study on single-port endoscopic resection via a gasless transaxillary approach in the treatment of breast fibroadenoma in adolescents
Source: BMC Surg. 2023 Sep 14;23:279. doi: 10.1186/s12893-023-02186-1 (PMC10503113; doi:10.1186/s12893-023-02186-1)
Supplement: Supplementary file 9 — Supplementary Material 9 [file 12893_2023_2186_MOESM9_ESM.pdf]

SPRINGER NATURE

Author Services

Editing Certificate

This document certifies that the manuscript

Clinical Study on Single-Port Endoscopic Resection via Gasless Transaxillary Approach in the Treatment of Breast Fibroadenoma in Adolescents

prepared by the authors

Jing-Yu Lu, Guo-Liang Zhang, Xiao-Jing Lin, Dar-Ren Chen, Zi-Fang Zheng, Yu Chen, Li-sheng Lin

was edited for proper English language, grammar, punctuation, spelling, and overall style by one or more of the highly qualified native English speaking editors at SNAS.

This certificate was issued on **August 27, 2023** and may be verified on the SNAS website using the verification code **93A2-FA14-82B0-D493-B156** .

Neither the research content nor the authors' intentions were altered in any way during the editing process. Documents receiving this certification should be English-ready for publication; however, the author has the ability to accept or reject our suggestions and changes. To verify the final SNAS edited version, please visit our verification page at secure.authorservices.springernature.com/certificate/verify.
If you have any questions or concerns about this edited document, please contact SNAS at support@as.springernature.com.

SNAS provides a range of editing, translation, and manuscript services for researchers and publishers around the world.
For more information about our company, services, and partner discounts, please visit authorservices.springernature.com.
