# Supplementary material for: Clinical study on single-port endoscopic resection via a gasless transaxillary approach in the treatment of breast fibroadenoma in adolescents
Source: BMC Surg. 2023 Sep 14;23:279. doi: 10.1186/s12893-023-02186-1 (PMC10503113; doi:10.1186/s12893-023-02186-1)

1.Enter the link and click the botton(Mark with arrows)


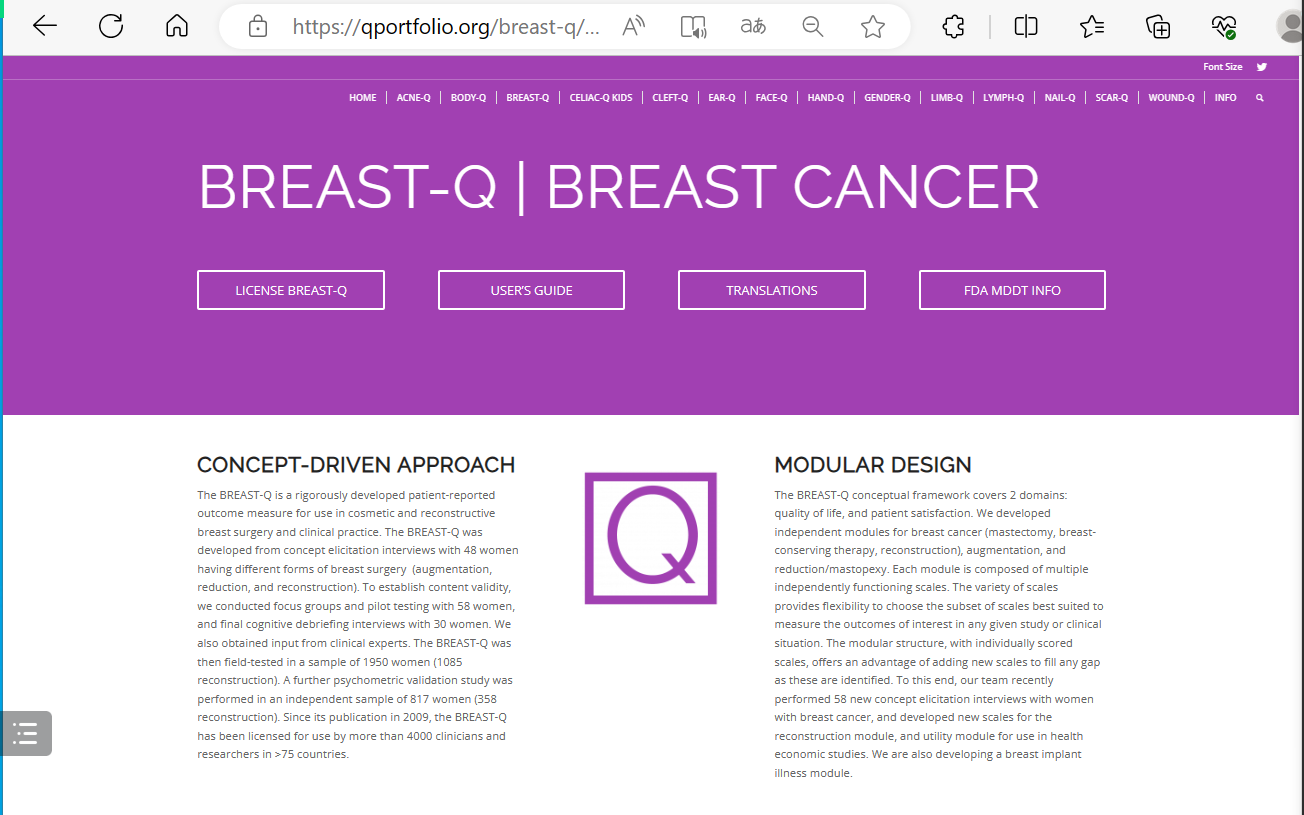


2.Select “yes”


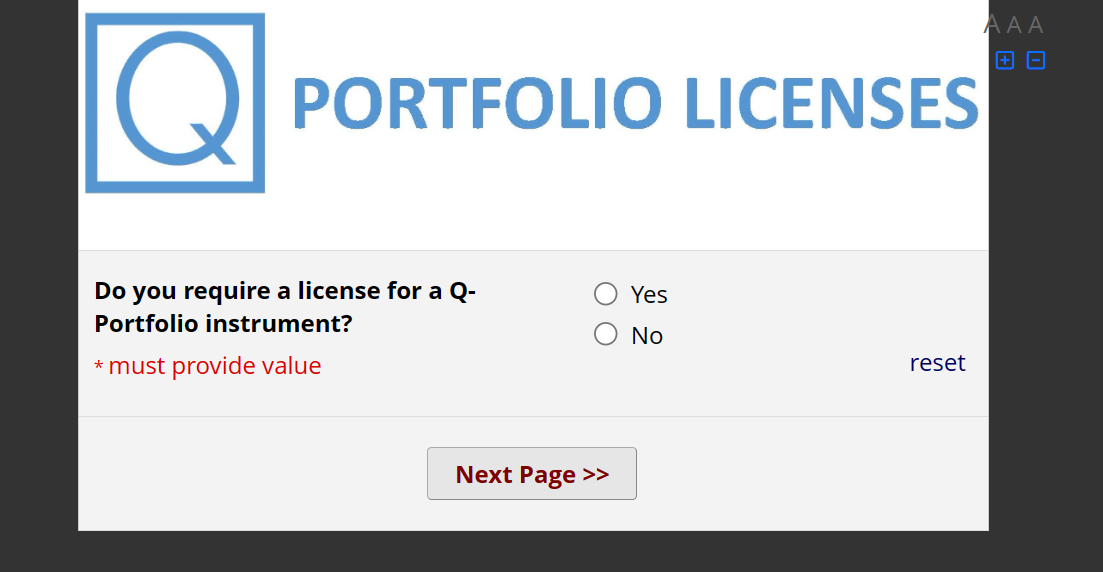


3.Select “Marked place”


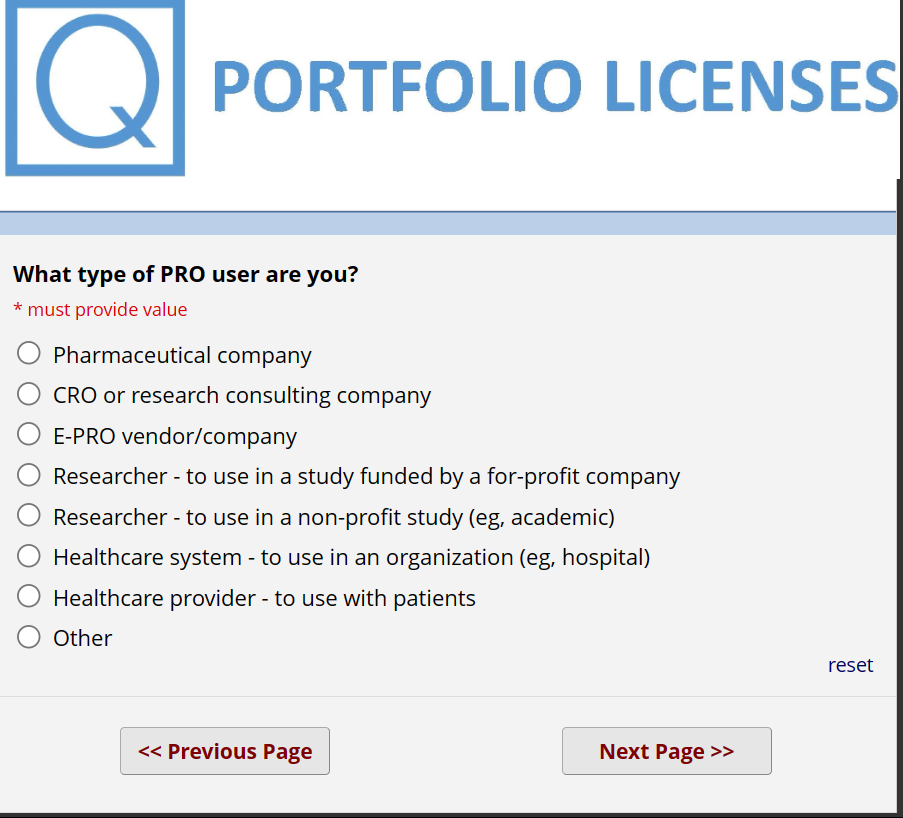


4.Select“BREAST-Q Breast Reduction/Mastopexy”


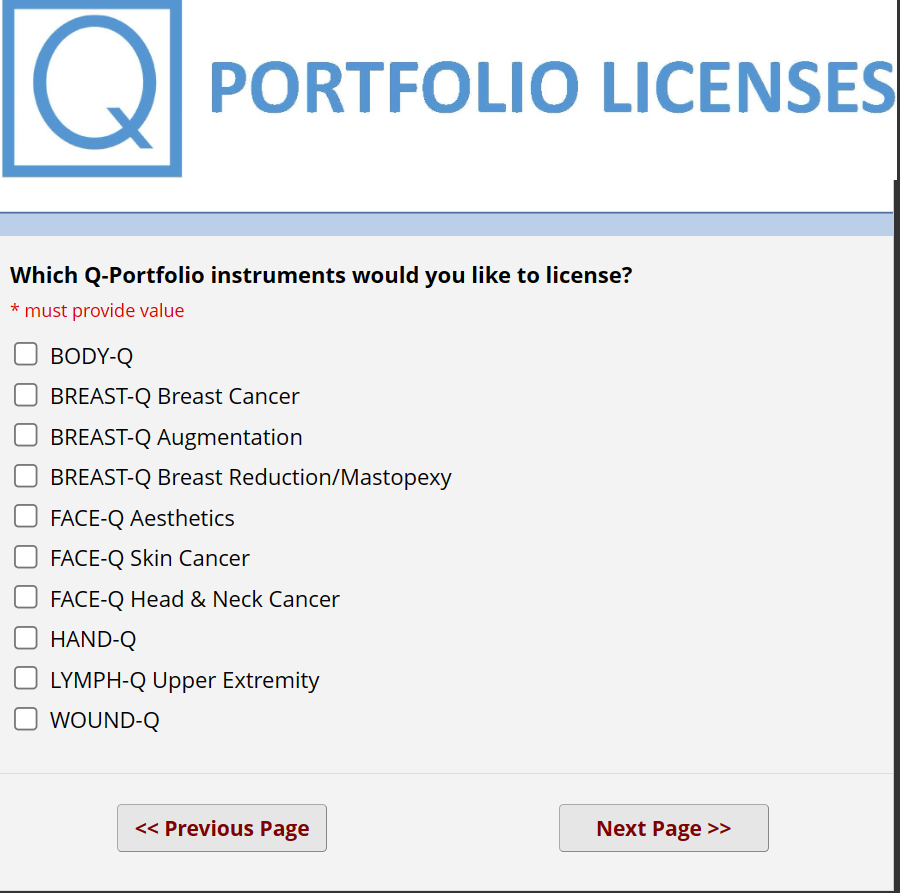


5.Select country name and provide email information


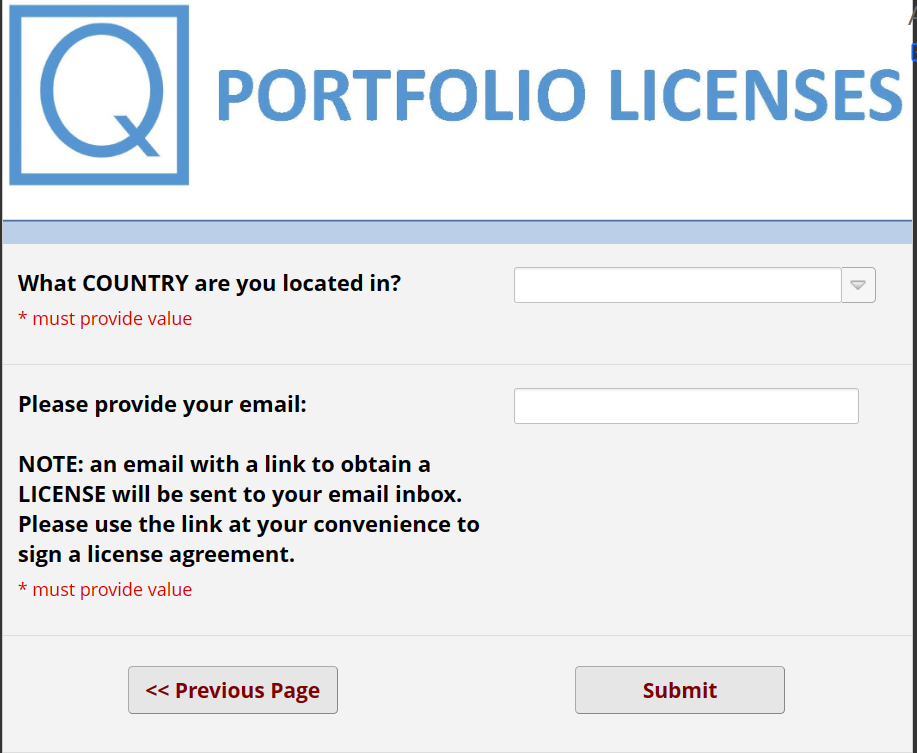


6.Click on the link in the received email


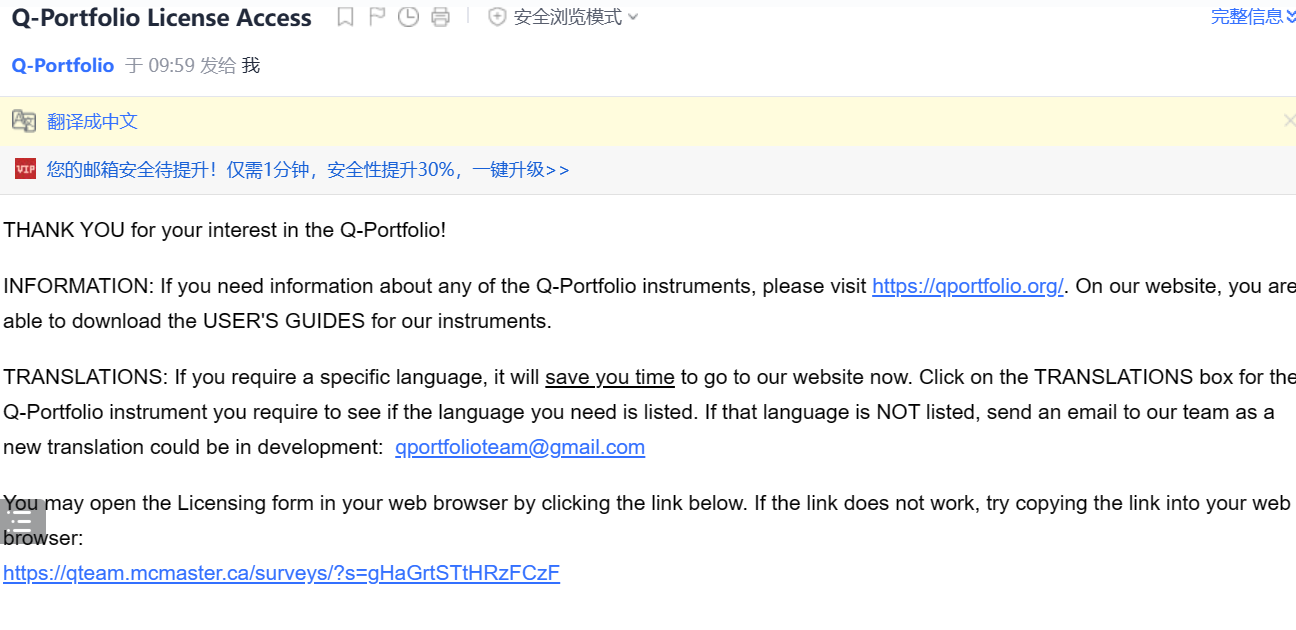


7.Go to the next step


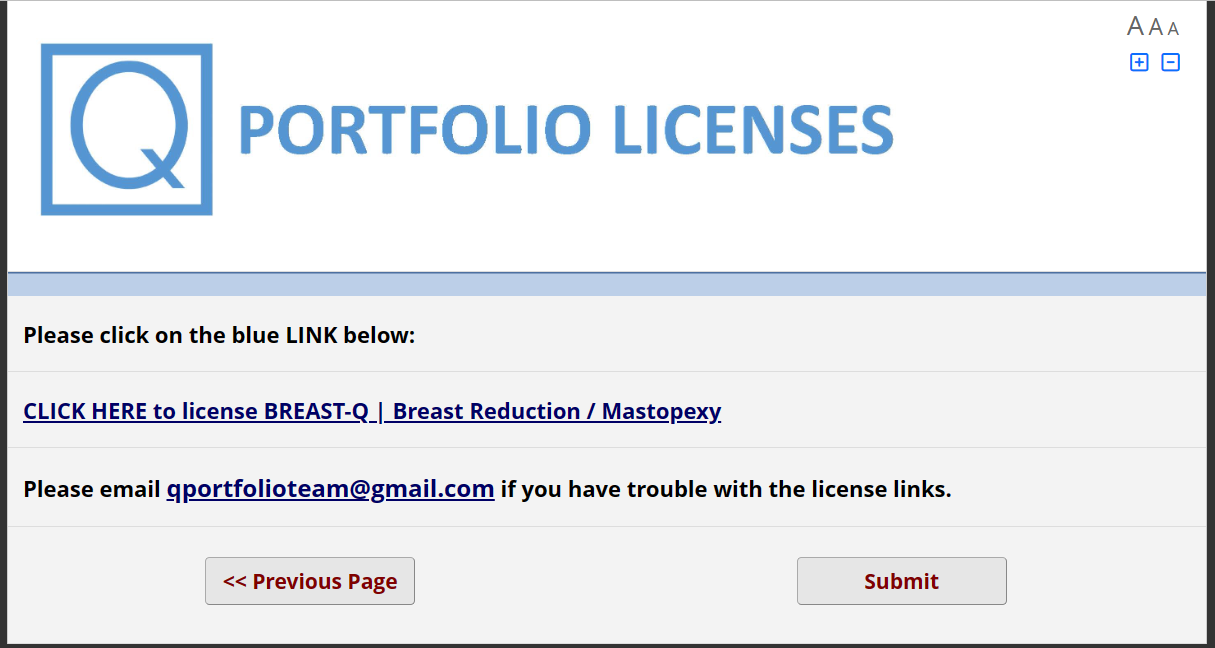


8.Select the corresponding option


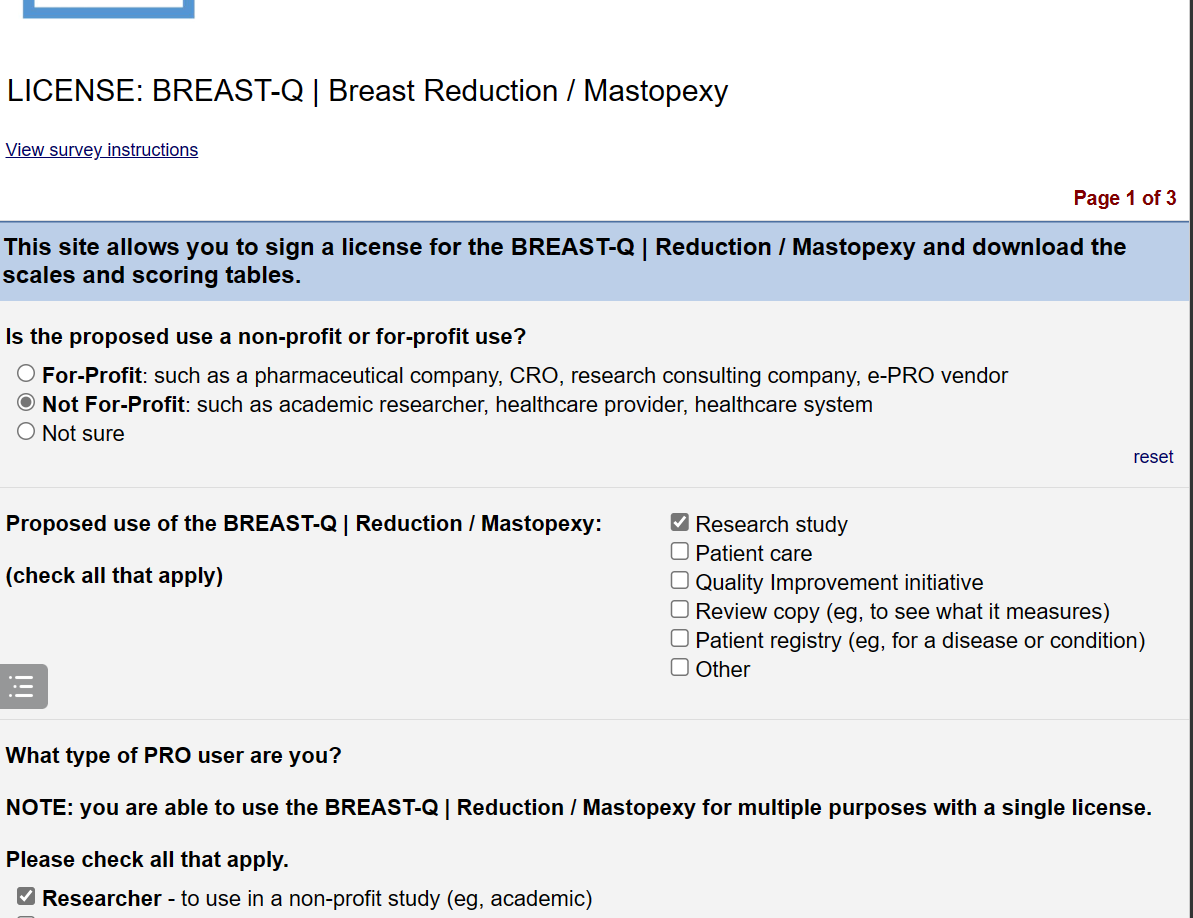


9.Select the corresponding option


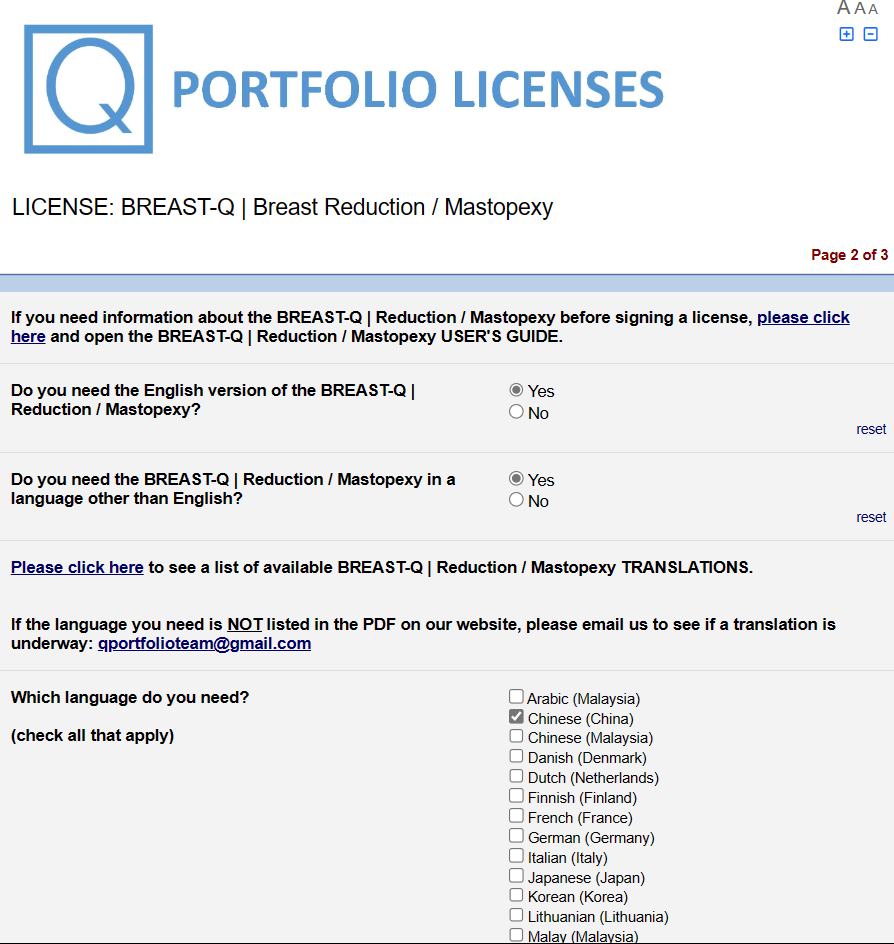


10.Fill in information


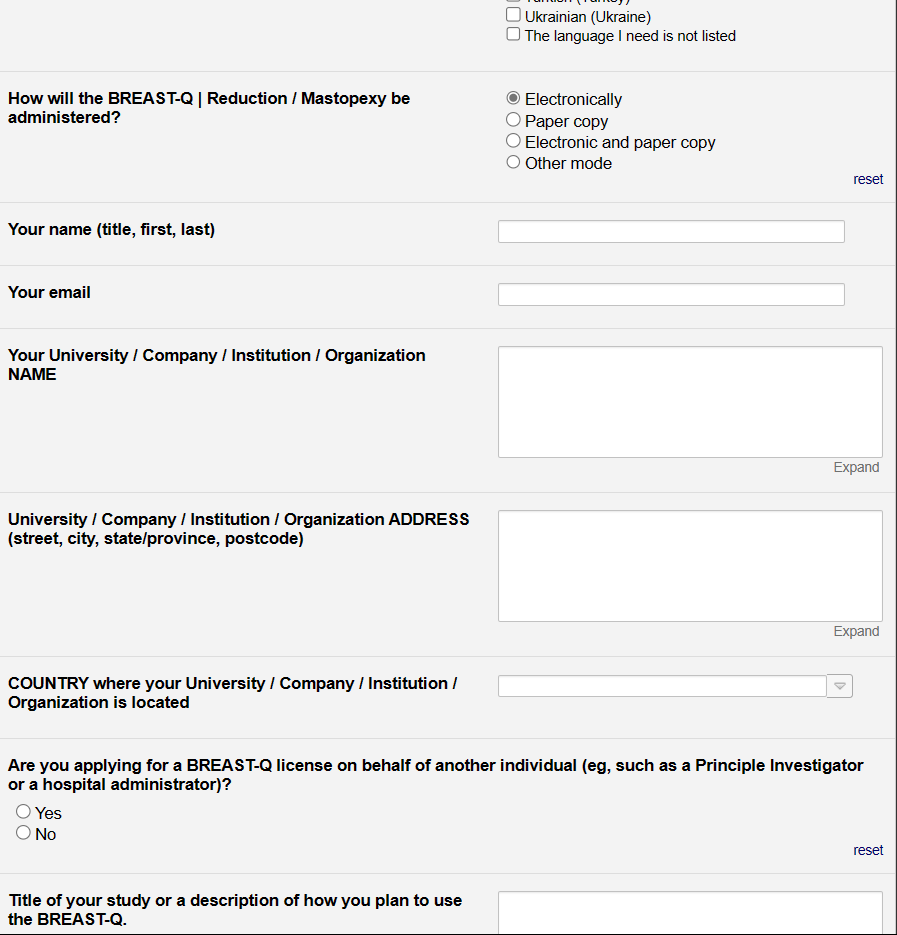


11.Download


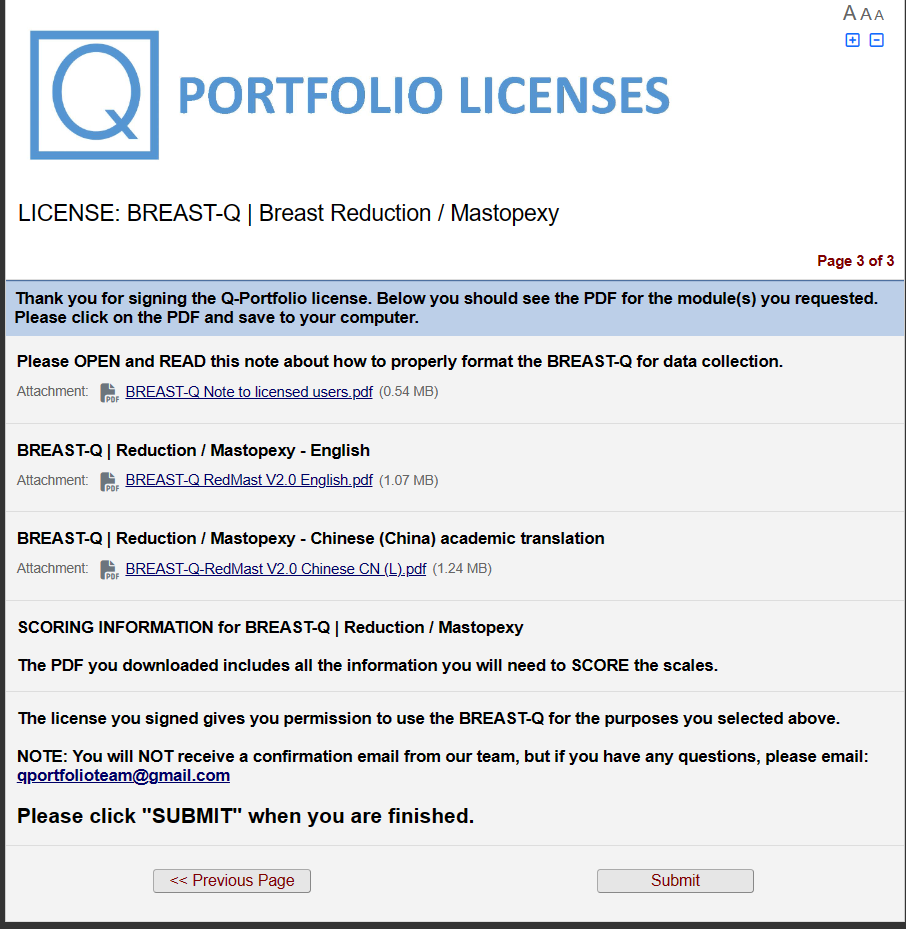

Supplement: Supplementary file 10 — Supplementary Material 10 [file 12893_2023_2186_MOESM10_ESM.docx]
